# Gene therapy with bidridistrogene xeboparvovec for limb-girdle muscular dystrophy type 2E/R4: phase 1/2 trial results

**DOI:** 10.1038/s41591-023-02730-9

**Published:** 2024-01-04

**Authors:** Jerry R. Mendell, Eric R. Pozsgai, Sarah Lewis, Danielle A. Griffin, Linda P. Lowes, Lindsay N. Alfano, Kelly J. Lehman, Kathleen Church, Natalie F. Reash, Megan A. Iammarino, Brenna Sabo, Rachael Potter, Sarah Neuhaus, Xiaoxi Li, Herb Stevenson, Louise R. Rodino-Klapac

**Affiliations:** 1 Center for Gene Therapy, The Research Institute at Nationwide Children’s Hospital, Columbus, OH USA; 2 Department of Pediatrics, The Ohio State University, Columbus, OH USA; 3 Department of Neurology, The Ohio State University, Columbus, OH USA; 4 Sarepta Therapeutics, Inc., Cambridge, MA USA

**Keywords:** Targeted gene repair, Neuromuscular disease

## Abstract

Limb-girdle muscular dystrophy 2E/R4 is caused by mutations in the β-sarcoglycan (*SGCB*) gene, leading to SGCB deficiency and consequent muscle loss. We developed a gene therapy approach based on functional replacement of the deficient SCB protein. Here we report interim results from a first-in-human, open-label, nonrandomized, phase 1/2 trial evaluating the safety and efficacy of bidridistrogene xeboparvovec, an adeno-associated virus-based gene therapy containing a codon-optimized, full-length human *SGCB* transgene. Patients aged 4–15 years with confirmed *SGCB* mutations at both alleles received one intravenous infusion of either 1.85 × 10^13^ vector genome copies kg^−^^1^ (Cohort 1, *n* = 3) or 7.41 × 10^13^ vector gene copies kg^−1^ (Cohort 2, *n* = 3). Primary endpoint was safety, and secondary endpoint was change in SGCB expression in skeletal muscle from baseline to Day 60. We report interim Year 2 results (trial ongoing). The most frequent treatment-related adverse events were vomiting (four of six patients) and gamma-glutamyl transferase increase (three of six patients). Serious adverse events resolved with standard therapies. Robust SGCB expression was observed: Day 60 mean (s.d.) percentage of normal expression 36.2% (2.7%) in Cohort 1 and 62.1% (8.7%) in Cohort 2. Post hoc exploratory analysis showed preliminary motor improvements using the North Star Assessment for Limb-girdle Type Muscular Dystrophies maintained through Year 2. The 2-year safety and efficacy of bidridistrogene xeboparvovec support clinical development advancement. Further studies are necessary to confirm the long-term safety and efficacy of this gene therapy. ClinicalTrials.gov registration: NCT03652259.

## Abstract (web summary)

Two-year interim data from an ongoing phase 1/2 trial of the AAV-based gene therapy bidridistrogene xeboparvovec in six patients with limb-girdle muscular dystrophy show a safe profile and preliminary increased expression of the β-sarcoglycan protein.

## Main

Limb-girdle muscular dystrophies (LGMDs) are a clinically and genetically diverse group of autosomal inherited monogenic neuromuscular diseases characterized by muscle wasting and progressive weakness, mostly within proximal muscles around the pelvic and shoulder girdles^1–3^. Sarcoglycanopathies are among the most severe LGMDs, with patients typically showing rapidly progressive proximal muscle weakness, respiratory impairment and potential cardiac abnormalities. LGMD type 2E/R4 (LGMD2E/R4) is caused by mutations in the β-sarcoglycan gene (*SGCB*), leading to β-sarcoglycan (SGCB) protein deficiency and loss of sarcoglycan complex formation and preventing dystrophin-associated protein complex (DAPC) stabilization^4,5^. This loss of protein function manifests clinically in early childhood or late adolescence and progresses to loss of ambulation, potential respiratory insufficiency and often premature mortality^6–8^.

Globally, the incidence of LGMD2E/R4 is between one in 200,000 and one in 350,000 (ref. ^9^). Diagnosis typically occurs before the age of 10 years, with loss of ambulation by middle to late teens^10–12^. Patients present with elevated serum creatine kinase (CK), difficulty rising from the floor or walking and proximal muscle weakness. Joint contractures and kyphoscoliosis, combined with degenerating diaphragmatic muscle, lead to compromised pulmonary function^13,14^. Both the need for assisted ventilation with disease progression and cardiac involvement occurring in about 60% of patients contribute to poor prognosis and premature death^6,13,15–18^. There is an urgent need for a viable treatment option, because there are currently no approved therapies. Because LGMD2E/R4 is a monogenic disorder and its impact is largely restricted to skeletal and cardiac muscle, it is particularly suited for adeno-associated virus (AAV)-mediated gene transfer therapy.

Evaluation of any gene therapy platform must include a systematic and stepwise consideration of safety, transduction, expression, localization, cellular impact and clinical function^19^. The rAAVrh74.MHCK7.hSGCB construct, referred to as bidridistrogene xeboparvovec (SRP-9003), is a self-complementary AAV vector designed with these considerations in mind, containing a codon-optimized, full-length human *SGCB* (h*SGCB*) transgene, and driven by a muscle-specific promoter (MHCK7) to restore functional SGCB protein in muscle cells that leads to functional improvements in patients with LGMD2E/R4. AAVrh74 is a rhesus monkey-derived AAV vector that shows robust skeletal and cardiac muscle tissue tropism^20,21^. The MHCK7 promoter drives robust transgene expression in both skeletal and cardiac muscle and limits expression in off-target tissues. The addition of the promoter region of α-myosin heavy chain enhances cardiac expression^20,22^. The h*SGCB* transgene carries full-length human *SGCB* cDNA. The self-complementary nature of bidridistrogene xeboparvovec allows it to overcome the rate-limiting step for AAVs of second-strand synthesis, resulting in more efficient transduction of target cells with rapid and higher transgene expression^23,24^.

In preclinical studies, bidridistrogene xeboparvovec gene transfer provided complete restoration of human SGCB protein expression with concomitant improvements in muscle histopathology, kyphoscoliosis, physical activity and cardiac and diaphragmatic function in mice, and no safety issues observed; specifically, diaphragm force production increased by 94% (*P* < 0.05) and ambulation by 57% (*P* < 0.05) following treatment compared with *SGCB*^−/−^ mice^20^.

**Fig. 1:**
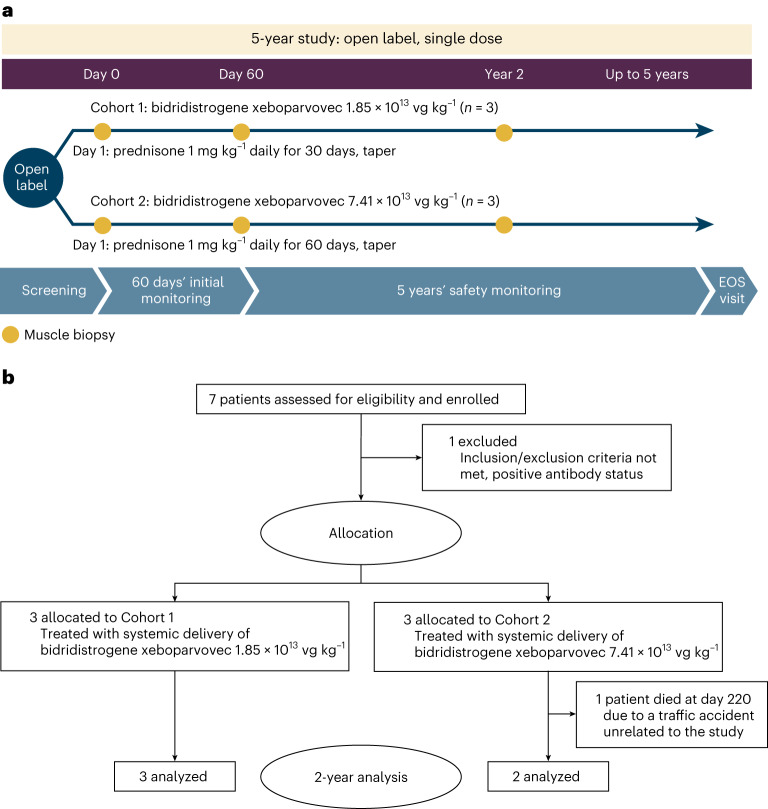
SRP-9003-101 study design and study flow diagram. **a**, Study design. **b**, Study flow diagram. EOS, end of study.

This report presents preliminary safety, tolerability and efficacy data of the first-in-human application of bidridistrogene xeboparvovec systemic gene delivery in patients with LGMD2E/R4, including up to 2 years of follow-up. The study includes two dose cohorts who received bidridistrogene xeboparvovec doses of either 1.85 × 10^13 ^vector genome copies (vg) kg^−1^ (Cohort 1) or 7.41 × 10^13 ^vg kg^−1^ (Cohort 2), calculated based on a validated quantitative polymerase chain reaction (qPCR) method using a linear standard (Fig. 1a).

## Results

### Patient baseline demographics and clinical characteristics

A total of seven patients were assessed for eligibility; of the six patients who met eligibility criteria, three each were enrolled in Cohorts 1 and 2 (Fig. 1b). The first patient was enrolled on 26 October 2018 (Cohort 1) and the last on 17 January 2020 (Cohort 2); the trial is ongoing. Five patients remain on study, and one patient died in an accident unrelated to the study on Day 220. Baseline demographics and clinical characteristics are shown in Table 1. All *SGCB* mutations are homozygous; five patients have missense mutations in exons 3 and 4, which encode for the extracellular domain of SGCB protein, and one patient has a nonsense mutation in exon 1; all of these mutations lead to the absence of SGCB protein and a severe phenotype^13^. The mean age at baseline was 10.0 years in both cohorts. For Cohorts 1 and 2, respectively, at baseline, mean weight was 42.3 and 32.1 kg, mean body mass index (BMI) was 20.5 and 17.4 and mean CK levels were 16,575.3 and 7,042.7 U l^−1^. Mean (s.d.) North Star Assessment for Limb-girdle Type Muscular Dystrophies (NSAD) score at baseline was 43.0 (4.4) points in Cohort 1 and 39.3 (2.1) points in Cohort 2.

**Table 1:** Baseline demographics and clinical characteristics

	Cohort 1				Cohort 2			
	Patient 1	Patient 2	Patient 3	Total^a^ (*n* = 3)	Patient 4	Patient 5	Patient 6	Total^a^ (*n* = 3)
Age, years	13	4	13	10.0 (5.2)	11	11	8	10.0 (1.7)
Sex^b^	Female	Male	Female	NA	Male	Male	Female	NA
Height, cm	155	103	157.5	138.5 (30.8)	132	144	129.2	135.1 (7.9)
Weight, kg	56.2	17.7	53.1	42.3 (21.4)	29.4	39.5	27.3	32.1 (6.5)
BMI, kg m^−^^2^	23.4	16.7	21.4	20.5 (3.4)	16.9	19	16.4	17.4 (1.4)
*SGCB* mutation^c^	Exon 3 c.341C>T; p.Ser114Phe Missense	Exon 4 c.452C>G; p.Thr151Arg Missense	Exon 3 c.341C>T; p.Ser114Phe Missense	NA	Exon 4 c.452C>G; p.Thr151Arg Missense	Exon 3 c.341C>T; p.Ser114Phe Missense	Exon 1 c.1_2delAT Deletion	NA
CK^d^, U l^−1^	10,727	28,014	10,985	16,575.3 (9907.0)	6,447	8,938	5,743	7,042.7 (1678.7)
Functional measures								
NSAD score	40	48	41	43.0 (4.4)	41	37	40	39.3 (2.1)
Time to rise, s	5	1.5	3.5	3.3 (1.8)	3.3	3.5	5.7	4.2 (1.3)
Four-stair climb, s	2.4	1.6	2.8	2.3 (0.6)	3	3.1	3.1	3.1 (0.1)
100-m, s	52	35.1	48.8	45.3 (9.0)	47.2	59.7	65.3	57.4 (9.3)
10-m, s	5	3.4	5.2	4.5 (1.0)	4.9	5.8	6.1	5.6 (0.6)

10-m, 10-m timed test; 100-m, 100-m timed test; BMI calculated as weight in kg divided by height in m^2^; NA, not applicable.

^a^Data expressed as mean (s.d.).

^b^Sex reported was determined based on self-report.

^c^Patients 1–5 had missense mutations; Patient 6 had a nonsense mutation; all mutations were homozygous.

^d^Baseline CK level defined as the maximum value of screening and Day −1 measurements; SI conversion factor: for conversion of U l^−1^ to μkat l^−1^, multiply by 0.0167.

### Safety and immunogenicity

The overall safety of bidridistrogene xeboparvovec was favorable across both cohorts at 2 years (Table 2 and Extended Data Table 1). In Cohort 1, two patients had treatment-related elevated γ-glutamyl transferase (GGT) levels that occurred either during or after tapering of oral steroids. One case was designated a serious adverse event (SAE) because the patient experienced AAV-related hepatitis and a transient increase in bilirubin. The SAE of hepatitis occurred on study Day 53, at the end of the steroid taper (patient was off steroids), and required short hospitalization; the event lasted 4 days and resolved with intravenous fluids and oral prednisone. In both cases, GGT levels returned to normal and symptoms were resolved within 30 days following supplemental steroid treatment. One patient experienced moderate upper gastrointestinal pain and mild vomiting that resolved within 1–2 days without steroid treatment. Other treatment-related adverse events were mild dizziness (*n* = 1) and mild decreased appetite (*n* = 1).

**Table 2:** Primary outcome of treatment-related, treatment-emergent adverse events by system organ class and preferred term at 2 years

System organ class preferred term	Cohort 1 (dose, 1.85 × 10^13 ^vg kg^−1^)^a^ (*n* = 3)	Cohort 2 (dose, 7.41 × 10^13 ^vg kg^−1^)^b^ (*n* = 3)	Total (*n* = 6)
Subjects with any treatment-related, treatment-emergent adverse event	2 (66.7%)	3 (100.0%)	5 (83.3%)
Gastrointestinal disorders	1 (33.3%)	3 (100.0%)	4 (66.7%)
Abdominal pain	0	2 (66.7%)	2 (33.3%)
Abdominal pain, upper	1 (33.3%)	1 (33.3%)	2 (33.3%)
Nausea	0	2 (66.7%)	2 (33.3%)
Vomiting	1 (33.3%)	3 (100.0%)	4 (66.7%)
General disorders and administration site conditions	0	1 (33.3%)	1 (16.7%)
Pyrexia	0	1 (33.3%)	1 (16.7%)
Hepatobiliary disorders	1 (33.3%)	0	1 (16.7%)
Hepatitis	1 (33.3%)^c^	0	1 (16.7%)
Hyperbilirubinemia	1 (33.3%)	0	1 (16.7%)
Investigations	2 (66.7%)	3 (100.0%)	5 (83.3%)
GGT increased	2 (66.7%)	1 (33.3%)	3 (50.0%)
Neutrophil count decreased	0	1 (33.3%)	1 (16.7%)
White blood cell count decreased	0	2 (66.7%)	2 (33.3%)
Metabolism and nutrition disorders	1 (33.3%)	1 (33.3%)	2 (33.3%)
Decreased appetite	1 (33.3%)	0	1 (16.7%)
Dehydration	0	1 (33.3%)^c^	1 (16.7%)
Nervous system disorders	1 (33.3%)	0	1 (16.7%)
Dizziness	1 (33.3%)	0	1 (16.7%)

Treatment-emergent adverse events are defined as all adverse events that started on or after the study drug administration date. Adverse events are coded using Medical Dictionary for Regulatory Activities v.22.0. Cutoff dates for safety data were 14 January 2021 (Cohort 1) and 18 January 2022 (Cohort 2).

^a^1.85 × 10^13 ^vg kg^−1^ (linear standard qPCR).

^b^7.41 × 10^13 ^vg kg^−1^ (linear standard qPCR).

^c^SAE.

In Cohort 2, most patients experienced mild-to-moderate treatment-related gastrointestinal adverse events (for example, vomiting, nausea, abdominal pain), all of which resolved. One patient had mildly elevated GGT levels, which returned to normal while tapering steroids; the patient did not experience an increase following tapering. One patient experienced severe dehydration resulting from vomiting 3 days following infusion, which was classified as a treatment-related SAE and required brief hospitalization; symptoms resolved in 2 days with ondansetron, promethazine and intravenous fluids. There was one death due to a traffic accident unrelated to the study. None of the protocol-specified conditions for halting the study (for example, unexpected, serious or unacceptable risk to patients) occurred, and there were no discontinuations or deaths related to the study.

**Extended Data Fig. 1:**
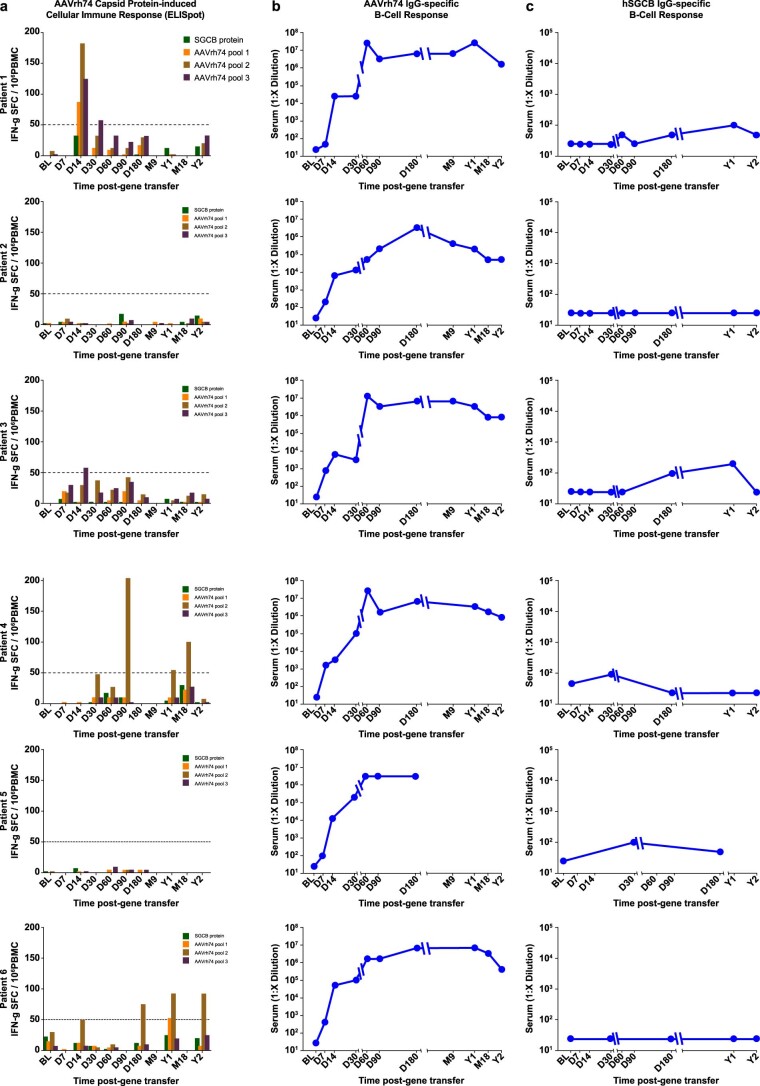
Exploratory outcomes of individual immune responses to AAVrh74 capsid protein, AAV peptide pools, and hSGCB. Exploratory outcomes of individual immune responses to (**a**) AAVrh74 capsid protein, (**b**) AAV peptide pools and (**c**) hSGCB. Footnote: Dotted line in ELISpot plots denotes threshold of 50 Spot Forming Colonies (SFC) per million cells. hSGCB, human β-sarcoglycan; IFN-γ SFC, gamma interferon spot-forming cells; PBMC, peripheral blood mononuclear cells. Missing datapoints are due to patient samples not collected at all or not collected at the scheduled timepoint (unscheduled data not shown). Asterisk (‘*’) denotes value <1/X. Hashtag (‘#’) denotes value >1/X.

There were no adverse immune responses and no clinically meaningful B or T cell responses toward AAVrh74 or SGCB protein (ELISPots) or increases in anti-SGCB antibodies (ELISA assay) in any patient (Extended Data Fig. 1). There were expected increases in AAVrh74 antibodies in all six patients post dosing (Extended Data Fig. 1). T cell response toward AAVrh74 peptide pools (1–3) demonstrated increases in three patients, with the stronger response observed for peptide pool 2 (Extended Data Fig. 1). Elevations did not correspond to liver enzyme elevation or impact transgene expression. Notably, none of the adverse events were related to clinical complement activation.

No other laboratory abnormalities were noted that were suggestive of safety concerns. No decreases in platelet counts outside the normal range or electrocardiogram abnormalities were observed.

### Bidridistrogene xeboparvovec transduction, SGCB expression and cell localization

**Extended Data Fig. 2:**
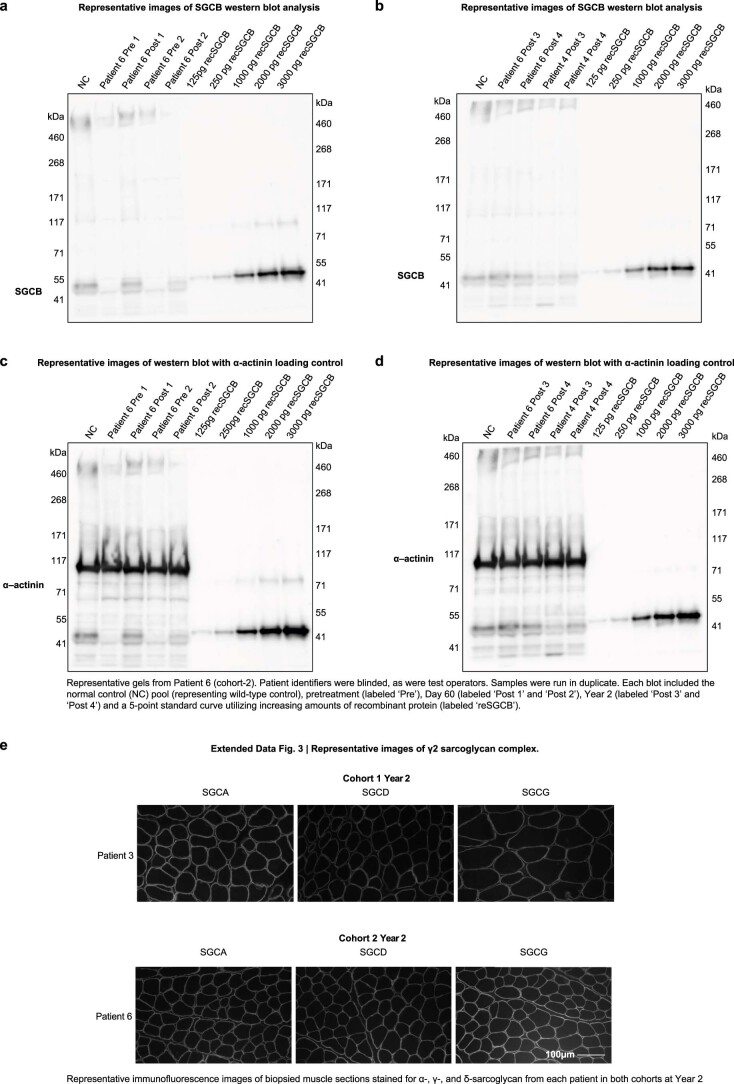
Secondary and exploratory outcomes. representative images of SGCB immunoblot analysis (**a**, **b**), immunoblot with α-actinin loading control (**c, d**), and γ2 sarcoglycan complex (**e**). Source data

Bidridistrogene xeboparvovec transduction and SGCB protein expression following systemic infusion were analyzed at two different timepoints (Day 60 and Year 2) and were detected by vector-specific droplet digital PCR (ddPCR), immunoblot and immunofluorescence (Table 3). Successful transduction in skeletal muscle (tibialis anterior and biceps) was confirmed in both cohorts at Day 60. In Cohort 1, data for vg per nucleus by ddPCR are not available; in Cohort 2, mean (s.d.) vg by ddPCR were 2.26 (0.92). Immunoblot analysis confirmed full-length SGCB protein expression in all patients, with a mean (s.d.) percentage of normal expression at Day 60 of 36.2% (2.7%) for Cohort 1 and 62.1% (8.7%) for Cohort 2. Representative immunoblot images of pretreatment (baseline), Day 60 and Year 2 are shown in Extended Data Fig. 2.

**Table 3:** Secondary and exploratory outcomes of SGCB expression in muscle biopsies (tibialis anterior and biceps) at Day 60 and Year 2

	Cohort 1 (dose, 1.85 × 10^13 ^vg kg^−1^)^a^ (*n* = 3)					Cohort 2 (dose, 7.41 × 10^13 ^vg kg^−1^)^b^ (*n* = 3)^c^				
Timepoint	Immunoblot (% NC)	Percentage SGCB^+^ fibers (% NC)	SGCB intensity (% NC)	Vector copies per nucleus		Immunoblot (% NC)	Percentage SGCB^+^ fibers (% NC)	SGCB intensity (% NC)	Vector copies per nucleus	
				qPCR	ddPCR				qPCR	ddPCR
Baseline mean (s.d.)	NA	8.9 (4.7)	7.4 (6.0)	0	NA	33.1 (4.8)	5.9 (6.4)	4.6 (5.7)	0	0
Median (range)	NA	8.5 (4.4, 13.7)	4.7 (3.3, 14.3)	0	NA	35.0 (27.6, 36.6)	5.0 (0, 12.8)	2.5 (0.2, 11.1)	0	0
Day 60 mean (s.d.)	36.2 (2.7)	51.0 (10.6)	47.4 (9.5)	0.6 (0.4)	NA	62.1 (8.7)	72.3 (6.2)	73.1 (21.8)	4.2 (2.8)	2.3 (0.9)
Median (range)	34.7 (34.6, 39.3)	48.9 (41.6, 62.5)	47.4 (38.0, 56.9)	0.4 (0.3, 1.1)	NA	63.1 (53.0, 70.3)	74.8 (65.3, 77.0)	67.1 (54.9, 97.3)	4.0 (1.6, 7.2)	2.4 (1.3, 3.1)
Year 2 mean (s.d.)	54.0 (16.1)	46.8 (21.3)	35.1 (22.9)	0.1 (0.1)	0.46 (0.36)	60.3 (21.4)	63.1 (21.6)	43.5 (33.2)	0.2 (0.1)	0.5 (0.3)
Median (range)	56.2 (37.0, 69.0)	58.6 (22.3, 59.7)	45.9 (8.8, 50.5)	0.1 (0.1, 0.2)	0.38 (0.2, 0.9)	60.3 (45.1, 75.4)	63.1 (47.9, 78.4)	43.5 (20.0, 67.0)	0.2 (0.2, 0.3)	0.5 (0.3, 0.8)

Values are mean (s.d.) and median (range); NC, normal control.

^a^1.85 × 10^13 ^vg kg^−1^ (linear standard qPCR).

^b^7.41 × 10^13 ^vg kg^−1^ (linear standard qPCR).

^c^*n* = 2 at Year 2.

**Fig. 2:**
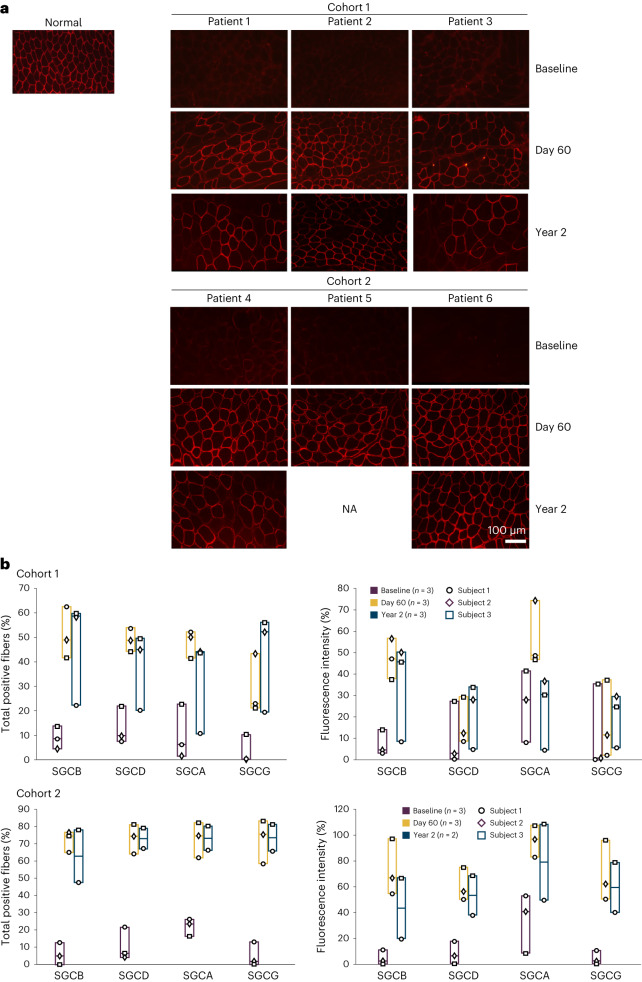
Secondary and exploratory outcomes of SGCB and sarcoglycan complex expression. **a**, Representative immunofluorescence images of biopsied muscle sections stained for SGCB from each patient pre and post treatment (Day 60 and Year 2) compared with normal muscle. Biopsied tissue sections of tibialis anterior and biceps were processed and stained for detection of SGCB. Four ×20 images per tissue block were taken, with four per timepoint (that is, 16 images per patient per timepoint). Patient 5 died in an accident unrelated to the study and therefore the Year 2 biopsy timepoint is missing. **b**, Expression of sarcoglycan complex subunits in Cohorts 1 and 2 from muscle biopsies by immunofluorescence (mean percentage of positive fibers and mean intensity) at baseline, Day 60 and Year 2.

Immunofluorescence staining was used to determine the percentage of positive fibers expressing SGCB protein and its correct localization in the sarcolemma in muscle tissue. SGCB protein was correctly localized to the sarcolemma at Day 60 (Fig. 2a), resulting in a mean (s.d.) percentage of SGCB-positive fibers of 51% (10.6%) and 72% (6.2%) in Cohorts 1 and 2, respectively. Protein expression was also quantified by fluorescence intensity at Day 60 (Table 3), resulting in a mean (s.d.) intensity of 47% (9.5%) and 73% (21.8%) of normal for Cohorts 1 and 2, respectively.

Bidridistrogene xeboparvovec vector genomes and protein expression in muscle tissue were durable, as seen in the analysis of muscle biopsies at Year 2 (Table 3). In Cohort 1, mean (s.d.) vg per nucleus by ddPCR were 0.46 (0.36); in Cohort 2, mean (s.d.) vg were 0.52 (0.33). SGCB protein expression by immunoblot was also sustained, with a mean (s.d.) percentage of normal expression at Year 2 of 54.0% (16.1%) for Cohort 1 and 60.3% (21.4%) for Cohort 2. At Year 2, mean (s.d.) percentage of SGCB-positive fibers was 47% (21.3%) and 63% (21.6%) in Cohorts 1 and 2, respectively, and mean (s.d.) intensity of normal expression was 35% (22.9%) and 44% (33.2%), respectively.

### Exploratory endpoint of restoration of DAPC

Lack of SGCB protein expression results in the mislocalization and downregulation of the sarcoglycan protein complex (composed of α-, γ- and δ-sarcoglycan (SGCA, SGCG and SGCD, respectively)) because it is not able to assemble correctly at the membrane without SGCB.

Immunofluorescence staining of the sarcoglycan complex subunits at both baseline and Day 60 demonstrated that SGCB protein expression corresponded to a clear improvement in the colocalization of SGCA, SGCG and SGCD subunits and rescue of the sarcoglycan complex at the membrane following treatment in both cohorts (Fig. 2b). At Day 60, both the percentage of fibers staining positive and fluorescence intensity increased for SGCA, SGCG and SGCD in patients of both cohorts, but increases were higher in Cohort 2 patients. At Year 2, the expression of sarcoglycan complex subunits remained higher than baseline in all patients and was generally maintained at similar expression levels compared with Day 60 (Fig. 2b). Individual patient data are shown in Supplementary Tables 1 and 2.

### Exploratory endpoint of CK reduction

Creatine kinase in serum was markedly elevated in patients with LGMD2E/R4 as a consequence of muscle fiber breakdown. At 60 days post treatment, serum CK levels were reduced in all patients (Extended Data Table 2); mean (s.d.) percentage change from baseline to Day 60 was −92.4% (9.8%) for Cohort 1 and −94.9% (4.5%) for Cohort 2. At 2 years post treatment, all patients in Cohorts 1 and 2 maintained reductions in serum CK levels (Extended Data Table 2); mean (s.d.) percentage change from baseline was −83.4% (4.7%) and −67.3% (18.2%), respectively.

### Exploratory endpoints of functional outcomes

**Extended Data Fig. 3:**
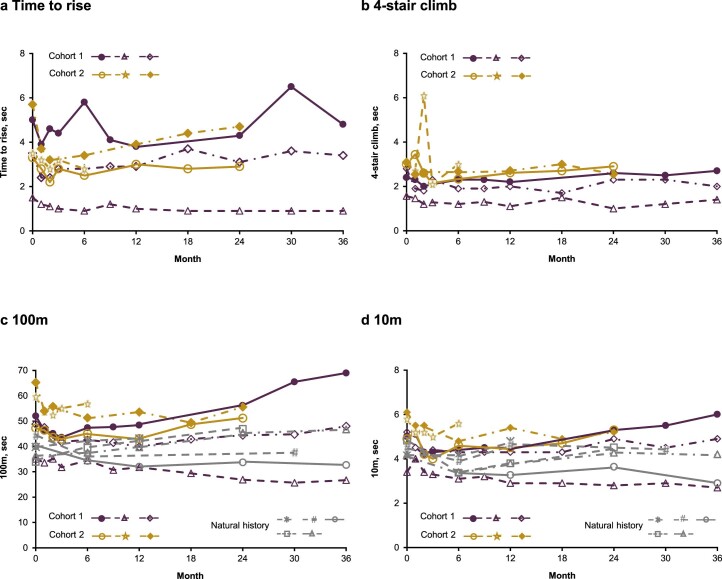
**Exploratory outcome of changes in functional outcomes throughout the study and post hoc comparison with natural history cohort presented as scores of individual patients for (a) Time to rise, (b) 4-stair climb, (c) 100m, (d) 10m**.

**Extended Data Fig. 4:**
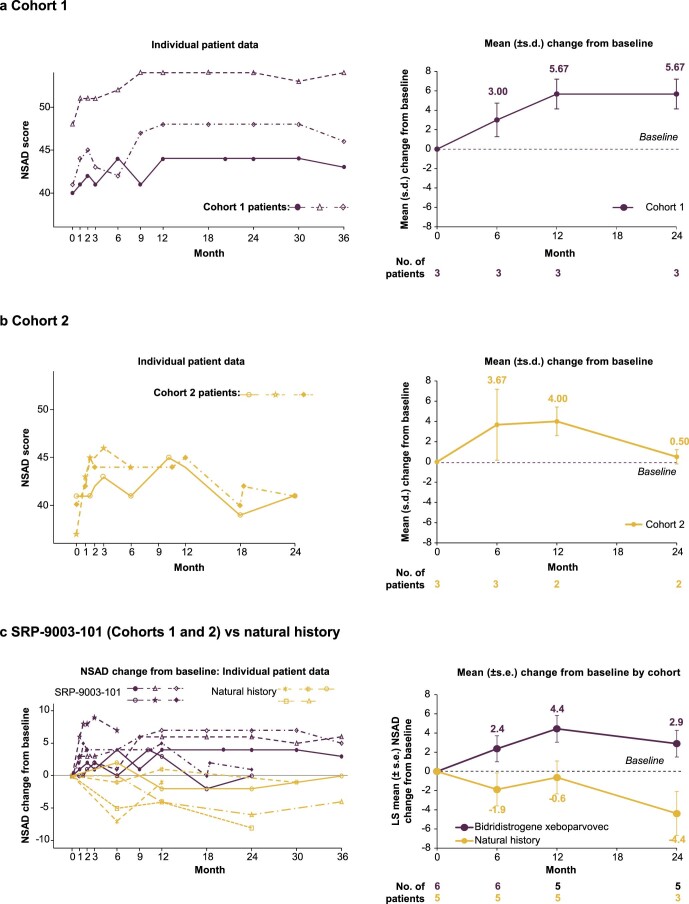
Exploratory outcome of changes in NSAD score throughout the study and post hoc comparison with natural history cohort. **a–c**, Changes in NSAD score in Cohort 1 (a), Cohort 2 (b), and SRP-9003-101 study population (Cohorts 1 and 2; *n* = 6) versus natural history cohort (*n* = 5) (c) presented as total NSAD score of individual patients (left panel) and mean (s.d.) change from baseline by cohort (right panel). The NSAD scale ranges from 0 to 54, with higher scores indicating a higher level of function. Footnote: ^a^MMRM analysis included fixed effects for treatment arm, visit, and treatment arm by visit interaction, and baseline NSAD, baseline 100m, and baseline 10m as continuous covariates; the first-order autoregressive structure was used for variance-covariance matrix of within-patient errors. Functional data for Patient 5 is available until month 6 due to a recreational accident unrelated to the study. LS, least square; MMRM, mixed model repeated measures; NSAD, North Star Assessment for Limb Girdle Type Muscular Dystrophies.

At 1 year post treatment, both cohorts showed improvements in functional outcomes (Table 4, Extended Data Fig. 3 and Extended Data Table 3). Motor performance was assessed using the NSAD scale; NSAD consists of 29 items, including running, squatting down and jumping (score range 0–54), with higher scores indicating higher levels of function. At 1 year, mean (s.d.) change from baseline in NSAD score was an increase of 5.7 (1.5) in Cohort 1 and an increase of 4.0 (1.4) in Cohort 2 (Extended Data Fig. 4). Timed function tests also improved at 1 year, with mean (s.d.) changes from baseline indicating shorter times for time to rise (Cohort 1, −0.8 s (0.4); Cohort 2, −1.1 s (1.1)); four-stair climb (Cohort 1, −0.5 s (0.3); Cohort 2, −0.4 s (0)); 100-m walk test (Cohort 1, −5.3 s (3.2); Cohort 2, −7.9 (5.4)); and 10-m walk test (Cohort 1, −0.6 s (0.2); Cohort 2, −0.6 s (0.2)).

**Table 4:** Exploratory outcomes of change from baseline in functional outcomes

	Cohort 1 (dose, 1.85 × 10^13 ^vg kg^−1^)^a^ (*n* = 3)			Cohort 2 (dose, 7.41 × 10^13 ^vg kg^−1^)^b^ (*n* = 3)			Natural history cohort (*n* = 5)		
	Month 6 (*n* = 3)	Year 1 (*n* = 3)	Year 2 (*n* = 3)	Month 6 (*n* = 3)	Year 1 (*n* = 2)	Year 2 (*n* = 2)	Month 6 (*n* = 5)	Year 1 (*n* = 5)	Year 2 (*n* = 5)
NSAD score									
Mean (s.d.)	3.0 (1.7)	5.7 (1.5)	5.7 (1.5)	3.7 (3.5)	4.0 (1.4)	0.5 (0.7)	–2.2 (3.7)	–2.0 (2.1)	–5.3 (3.1)
Median (range)	4.0 (1.0, 4.0)	6.0 (4.0, 7.0)	6.0 (4.0, 7.0)	4.0 (0, 7.0)	4.0 (3.0, 5.0)	0.5 (0, 1.0)	−1.0 (−7.0, 2.0)	−2.0 (−4.0, 1.0)	−6.0 (−8.0, −2.0)
Time to rise, s							–	–	–
Mean (s.d.)	−0.2 (0.8)	−0.8 (0.4)	−0.6 (0.2)	−1.3 (0.9)	−1.1 (1.1)	−0.7 (0.4)			
Median (range)	−0.6 (−0.7, 0.8)	−0.6 (−1.2, −0.5)	−0.6 (−0.7, −0.4)	−0.8 (−2.3, −0.7)	−1.1 (−1.8, −0.3)	−0.7 (−1.0, −0.4)			
Four-stair climb, s							–	–	–
Mean (s.d.)	−0.5 (0.4)	−0.5 (0.3)	−0.3 (0.4)	−0.4 (0.3)	−0.4 (0)	−0.3 (0.3)			
Median (range)	−0.4 (−0.9, −0.1)	−0.5 (−0.8, −0.2)	−0.5 (−0.6, 0.2)	−0.4 (−0.7, −0.1)	−0.4 (−0.4, −0.4)	−0.3 (−0.5, −0.1)			
100-m walk, s									
Mean (s.d.)	−3.8 (2.9)	−5.3 (3.2)	−2.8 (6.4)	−6.3 (6.7)	−7.9 (5.4)	−2.9 (9.7)	−0.8 (5.6)	1.6 (6.8)	5.2 (10.2)
Median (range)	−4.6 (−6.2, 0.6)	−3.6 (−8.9, −3.3)	−4.4 (−8.2, 4.2)	−2.8 (−14.0, −2.1)	−7.9 (−11.7, −4.1)	−2.9 (−9.7, 4.0)	1.2 (−7.5, 5.5)	3.3 (−8.1, 7.7)	9.0 (−6.4, 13.0)
10-m walk, s									
Mean (s.d.)	−0.6 (0.3)	−0.6 (0.2)	−0.2 (0.5)	−0.6 (0.6)	−0.6 (0.2)	−0.3 (0.9)	−0.7 (0.6)	−0.2 (0.7)	−0.1 (0.3)
Median (range)	−0.6 (−0.9, −0.3)	−0.5 (−0.9, −0.5)	−0.3 (−0.6, 0.3)	−0.3 (−1.3, −0.2)	−0.6 (−0.7, −0.4)	−0.3 (−0.9, 0.4)	−0.7 (−1.5, 0)	−0.1 (−1.0, 0.6)	−0.1 (−0.3, 0.1)

A positive (+) change in NSAD and a negative (−) change in timed tests indicate improvement in function.

^a^1.85 × 10^13 ^vg kg^−1^ (linear standard qPCR).

^b^7.41 × 10^13 ^vg kg^−1^ (linear standard qPCR).

Improvements over baseline in functional outcomes were generally sustained at 2 years in both cohorts (Table 4, Extended Data Fig. 3 and Extended Data Table 3). Mean (s.d.) change from baseline in NSAD score at 2 years was 5.7 (1.5) in Cohort 1 and 0.5 (0.7) in Cohort 2 (Extended Data Fig. 4). Mean (s.d.) changes from baseline in timed function tests at 2 years also indicated sustained improvements: time to rise (Cohort 1, −0.6 s (0.2); Cohort 2, −0.7 s (0.4)); four-stair climb (Cohort 1, −0.3 s (0.4); Cohort 2, −0.3 s (0.3)); 100-m test (Cohort 1, −2.8 s (6.4); Cohort 2, −2.9 s (9.7)); and 10-m test (Cohort 1, −0.2 s (0.5); Cohort 2, −0.3 s (0.9)). Hand-held dynamometry assessments showed sustained improvements at 2 years in six of six muscle groups in Cohort 1 and in four of six muscle groups in Cohort 2 (Extended Data Table 3).

### Post hoc comparison with natural history cohort

An exploratory comparison of functional outcomes in the SRP-9003-101 study population of bidridistrogene xeboparvovec-treated patients (Cohorts 1 and 2, *n* = 6) versus an untreated natural history cohort was also performed. The natural history cohort (*n* = 5) was identified from the Nationwide Children’s Hospital (NCH) natural history dataset using the same key study inclusion criteria as in SRP-9003-101 (further details provided in Methods). Whereas age and sex were well balanced between cohorts, the NCH natural history cohort had better functions as measured by NSAD, 100-m test and 10-m test at baseline (Extended Data Table 4). Alternative matching criteria were explored but were unsuccessful in achieving balanced baseline functions due to the limited sample size. A mixed-model, repeated-measures analysis was thus used to adjust for differences in baseline functions. The mixed-model, repeated-measures model included fixed effects for treatment arm, visit and treatment arm by visit interaction, as well as baseline NSAD, baseline 100-m test and baseline 10-m test as continuous covariates; the first-order autoregressive structure was used for variance–covariance matrix of within-patient errors; the Kenward–Roger approximation was used to estimate the denominator degrees of freedom. Individual patient-level data for the matched natural history cohort are shown in Extended Data Table 5.

In this exploratory comparison, patients treated with bidridistrogene xeboparvovec consistently showed higher NSAD change from baseline scores compared with the natural history cohort over 2 years of follow-up (Extended Data Fig. 4); least-squares mean difference in the change in NSAD score from baseline for the bidridistrogene xeboparvovec-treated cohort versus the natural history cohort at Year 2 was 7.3 (95% confidence interval (CI), 0.7–13.9). Timed function tests also showed improvements, with least-squares mean differences between cohorts in change from baseline of −6.2 s (95% CI, −19.6 to 7.1) for 100-m test and −0.8 s (95% CI, −2.2 to 0.6) for 10-m test. In addition, descriptive statistics are provided in Extended Data Table 6. The unadjusted mean difference in the change in NSAD score from baseline for the bidridistrogene xeboparvovec-treated cohort versus the natural history cohort at Year 2 was 8.9 (95% CI, 3.5–14.4). Timed function tests also showed improvements, with unadjusted mean differences between cohorts in change from baseline of −8.1 s (95% CI, −22.3 to 6.2) for 100-m test and −0.1 s (95% CI, −1.2 to 1.0) for 10-m test.

## Discussion

Interim results of this open-label trial provide biological and clinical evidence for the safety and efficacy of systemic gene therapy with bidridistrogene xeboparvovec, at both low and high doses, in patients with LGMD2E/R4.

Efficient transduction with widespread and robust full-length SGCB expression in muscle biopsies was shown by vector genome counts, immunofluorescence and immunoblot in all six patients at Day 60 post treatment, demonstrating success of the targeted delivery of the transgene to muscle tissue. Greater effects on transduction and SGCB expression were observed in the higher-dose cohort, indicating a dose–response in biological activity between low and high doses of bidridistrogene xeboparvovec. Immunofluorescence analysis showed that SGCB expression was associated with upregulation of SGCA, SGCG and SGCD expression, suggesting that bidridistrogene xeboparvovec can restore the sarcoglycan complex at the sarcolemma, which is required for DAPC stabilization. SGCB gene transduction and protein expression were also analyzed 2 years post treatment. We observed a reduction in SGCB genome copies compared with Day 60, which could be related to a higher amount of episomal DNA in the nucleus in the first months post gene transference. The initial overestimation of copy number was corrected by episomal DNA stabilization, which in turn provided a path for maintaining transgene expression and protein production at high levels, as measured by immunoblot and the percentage of SGCB-positive fibers. We observed a reduction in immunofluorescence intensity of SGCB at year 2 that was more evident in Cohort 1, but expression levels were comparable with Day 60.

These findings follow reports of delandistrogene moxeparvovec gene transfer therapy in Duchenne muscular dystrophy^25,26^. The construct used in the current study contains the same vector and promoter used in delandistrogene moxeparvovec, demonstrating consistent ability to drive transgene expression and consequent functional benefit in multiple disease states. Thereby, efficient and targeted production of native SGCB protein in the current trial resulted in the functional benefits observed.

Systemic administration of bidridistrogene xeboparvovec was well tolerated, with minimal SAE findings and no unexpected immunological responses. Some patients in both cohorts had elevated liver enzymes, but no associated clinical manifestations were observed. One SAE was observed in each cohort; in both cases, symptoms resolved with standard therapies and no stopping/discontinuation rules were triggered by these adverse events. Importantly, clinically relevant complement activation reported in gene transfer programs using other vectors (that is, non-AAVrh74) was not observed, which is in line with other AAVrh74-based programs in development.

This study has the inherent limitations of a small, open-label trial with an interim, short-term analysis that limits the ability to draw inferences from secondary, exploratory and subgroup analyses, and highlights the need for longer follow-up to assess the safety and efficacy of the therapy. Moreover, variability associated with the immunofluorescence technique, and the use of an external comparator (natural history cohort) that was matched by age with the treatment cohorts but not by baseline NSAD, pose additional limitations. Despite these limitations, patients in both cohorts treated with bidridistrogene xeboparvovec demonstrated improvement or stabilization of NSAD scores from baseline up to 2 years. Functional gains were accompanied by sustained reductions in serum CK levels, indicating less muscle damage, although early reductions were probably due to prophylactic treatment of prednisone that was continued post treatment for at least 30 and 60 days for Cohorts 1 and 2, respectively. Exploratory post hoc analyses further showed that bidridistrogene xeboparvovec-treated patients had clinically important improvements in functional outcomes compared with a natural history cohort over 2 years of follow-up. A recent observational, retrospective study of a large cohort of patients with sarcoglycanopathies identified residual mutated protein expression levels <30% as an independent risk factor for loss of ambulation before 18 years of age^27^. In our LGMD2E/R4 trial, both low- and high-dose cohorts showed full wild-type protein production with a mean percentage of normal expression of 36.2 and 62.1, respectively, at Day 60, suggesting that sufficient SGCB expression levels were achieved and predictive of improved functional outcomes.

An important concern for gene transfer therapy is the potential for waning of effects over time. Until there is broader and longer-term clinical experience with gene therapies for multiple disease-state applications, we can only speculate on long-term efficacy. Long-term follow-up of two clinical trials involving 12 patients with spinal muscular atrophy type 1 (up to 7 years) and four patients with Duchenne muscular dystrophy (up to 4 years), treated with a dose range of AAV-based vectors similar to that in the present study, showed therapeutic efficacy sustained over time^26,28^. Assessment of long-term safety and efficacy from a small cohort of patients with LGMD2E/R4 is challenging, but the present trial yielded encouraging results for bidridistrogene xeboparvovec for at least 2 years; extended follow-up of patients is currently ongoing, with the final study analysis planned at 5 years.

## Methods

### Study design and participants

This first-in-human, single-center, nonrandomized, open-label, phase 1/2 systemic gene delivery trial (SRP-9003-101) evaluated the safety, tolerability and efficacy of bidridistrogene xeboparvovec in patients with LGMD2E/R4 (ClinicalTrials.gov identifier: NCT03652259; Fig. 1a). This trial was conducted at NCH (Columbus, OH), preregistered on 28 August 2018 (NCT03652259) and began on 26 October 2018. Although the duration of follow-up is 5 years, the protocol is currently amended to extend the study for an additional 2 years with a total of 7 years. This study is being conducted in accordance with the principles of the Declaration of Helsinki and the International Council for Harmonisation Good Clinical Practice guidelines^29^. The study protocol was approved by the NCH Institutional Review Board. Parents or legal guardians of all patients provided written informed consent before study participation and genetic testing and consented to disclosure of their demographics for publication purposes. There was no compensation for participation in the study other than covering for meals and travel-related expenses.

### Vector description

SRP-9003 is a self-complementary, nonreplicating, recombinant AAVrh74 vector containing codon-optimized, full-length SGCB cDNA under the control of the myosin heavy-chain/muscle CK (MHCK7) promoter (the full sequence is given in Supplementary Information)^20,30^. The vector cassette contains minimal elements required for gene expression, including AAV2 inverted terminal repeats, hSGCB, SV40 chimeric intron and synthetic polyadenylation site, all under the control of the MHCK7 promoter (Supplementary Fig. 1). The amino acid sequence of the SGCB protein produced from the SRP-9003 transgene is identical to that of normal wild-type hSGCB protein.

### Outcomes

The primary endpoint was to evaluate the safety of bidridistrogene xeboparvovec. The secondary endpoint was the change in quantity of SGCB protein in skeletal muscle from baseline to Day 60 as assessed by immunoblot and immunofluorescence staining analysis (fiber intensity and percentage of SGCB-positive fibers). Exploratory endpoints included functional outcomes (NSAD score and time function tests), skeletal muscle strength, serum CK levels, immunogenicity of bidridistrogene xeboparvovec and durability of SGCB expression in skeletal muscle. Because patients are now beyond the post-dosing interval where safety events are most likely to be attributed to gene therapy, and all protocol-defined muscle biopsies used to evaluate SCGB expression have been completed, this report was generated to provide preliminary results for the primary and secondary endpoints. A subsequent clinical report of the final 5-year data will be warranted after final study completion.

Eligible patients were aged 4–15 years (inclusive) with confirmed *SGCB* gene mutations for SGCB deficiency at both alleles. Additional inclusion criteria were demonstrable muscle weakness and ≥40% of normal 100-m test predicted for age-, height- and weight-matched healthy controls at the screening visit^31^. Sex or gender was not considered in the study design. Sex was self-reported by participants. Full eligibility criteria are as follows.

Subject inclusion criteria:

1. Subjects aged 4–15 years, inclusive
2. Males or females of any ethnic group
3. SGCB DNA gene mutations at both alleles (if genetic testing was completed at a laboratory that is not Clinical Laboratory Improvement Amendments certified, the testing may be repeated at the discretion of the principal investigator)
4. Weakness demonstrated, based on history of difficulty running, jumping and climbing stairs
5. 100-meter walk/run test result: ≥40% of that predicted for age-, height-, gender- and weight-matched healthy controls at the screening visit
6. Ability to cooperate with muscle testing
7. Willingness of sexually active subjects with reproductive capacity to practice reliable method of contraception (if appropriate) during the first 6 months following gene therapy

Subject exclusion criteria:

1. Active viral infection based on clinical observations
2. Cardiac MRI-determined left ventricular ejection fraction <40% 
3. Serological evidence of infection by human immunodeficiency virus, hepatitis B or hepatitis C
4. Diagnosis of (or ongoing treatment for) an autoimmune disease
5. Abnormal laboratory values considered clinically significant
6. Concomitant illness or requirement for chronic drug treatment that, in the opinion of the principal investigator, creates unnecessary risks for gene transfer
7. Pregnancy
8. With AAVrh74-binding antibody titers >1:400 as determined by ELISA; if endpoint titer is positive at screening, testing may be repeated in 1 month
9. Has a medical condition or circumstance that could compromise the protocol compliance or compromise safety
10. Severe infection (for example, pneumonia, pyelonephritis or meningitis) within 4 weeks preceding gene transfer visit (enrollment may be postponed)
11. Family does not want to disclose subject’s study participation with primary care physician or other medical providers

### Study treatment

Enrolled patients received a single systemic infusion of bidridistrogene xeboparvovec via a peripheral vein over approximately 1–2 h. Twenty-four hours preceding gene delivery, prophylactic oral prednisone (1 mg kg^−1^) or comparable glucocorticoid was administered and continued daily for 30 days for Cohort 1. In response to a treatment-related SAE of hepatitis in one patient, the data safety monitoring board approved a protocol amendment to specify administration of immunosuppression for at least 60 days for Cohort 2. In both cohorts this was followed by a tapering regimen based on the individual’s immune response to gene transfer as assessed by both ELISpot and liver function monitoring of GGT. Patients in Cohort 1 were treated with bidridistrogene xeboparvovec at a dose of 1.85 × 10^13 ^vg kg^−1^ (calculated by qPCR using a linear reference standard). This dose was selected with the goal of achieving ≥20% of normal expression levels extrapolated from preclinical studies correlating expression and function^20^. Muscle biopsies at Day 60 determined the dosage level for Cohort 2. According to the protocol adopted, because the number of muscle fibers expressing SGCB was <50% of normal control intensity in one or more of the treated patients, the bidridistrogene xeboparvovec dose was escalated to 7.41 × 10^13 ^vg kg^−1^ by linear qPCR (standard equivalent of 2 × 10^14 ^vg kg^−1^) following review and endorsement by the Data Safety Monitoring Board. Patients in Cohort 2 received bidridistrogene xeboparvovec at least 4 weeks post biopsy of Cohort 1.

### Safety assessments

Safety was assessed throughout the study by monitoring of vital signs, physical examinations, electrocardiograms, incidence of treatment-emergent adverse events and treatment-emergent SAEs and select laboratory assessments including hematological analysis, serum chemistry panels (liver function tests and CK level) and urinalysis. Patients were assessed at baseline and monitored on Days 1, 7, 14, 30, 45, 60, 90, 180 and 270 and Years 1, 1.5 (Month 18), 2, 2.5 (Month 30), 3, 4 and 5.

Immunological response was assessed by measurement of antibodies (ELISA) and T cell responses (ELISpot) to rAAVrh74 and/or SGCB (ELISA) at baseline, Days 7, 14, 30, 60, 90, 180 and 270 and Years 1, 1.5 and 2.

### Biological assessments

Muscle biopsies were performed at baseline, Day 60 and Year 2 on the tibialis anterior and biceps muscles, with appropriate anesthesia. Biopsies were performed by an interventional radiologist guided by ultrasound and using a 10-g Vacora biopsy probe^32^. Immunoblot assays were executed according to validated methodology adapted from Charleston et al.^33^. Muscle biopsy tissue was mounted with minimal optimal cutting temperature compound, gum tragacanth medium and/or Cryo-Gel, cryosectioned and allocated for analysis by immunoblot, qPCR, ddPCR and immunofluorescence. qPCR was used initially for analysis of vg numbers before switching to ddPCR as the preferred method. SGCB levels of samples were calculated from a five-point standard curve using full-length recombinant SGCB protein ranging from 125 to 2,000 pg. The protocol was modified before Cohort 2 sample analysis, expanding the standard curve range from 125 to 3,000 pg and ranging from 5 to 80%. Reported SGCB levels were the average value of both biological replicates and two technical gel replicates for each sample result. Mean values for each patient were obtained from four tissue blocks. Recombinant protein was used to generate a standard curve for quantitative analysis. BIOQUANT automated software was used to quantify the level of SGCB expression, and ImageJ NIH software^34^ to determine the number of muscle fibers expressing SGCB.

### ELISpot assay

Whole-blood samples were collected at intervals as defined by the adopted protocol. Peripheral blood mononuclear cells (PBMCs) were separated from whole blood by density-gradient centrifugation. Briefly, fresh blood samples were spun in a tabletop centrifuge (Sorvall Legend RT, Marshall Scientific) and the top plasma layer was removed and stored at −80 °C. The remaining blood was mixed with PBS^−/−^ (Invitrogen) underlayed with Ficoll-Paque (Cytiva) and spun for density-gradient separation. The middle layer containing PBMCs was removed and subjected to various wash–spin cycles before final resuspension in human AIM V medium (Invitrogen) supplemented with human antibody serum. PBMCs were then plated in IP filter plates (MilliporeSigma) at a density of 2 × 10^5^ cells per well, except for the positive control wells that were plated at 2.5 × 10^4^ cells per well. IP plate wells were precoated with monoclonal antibody provided with the human interferon-γ ELISpot kit (U-Cytech Biosciences). Three peptide pools were used for the AAVrh74 capsid protein (Genemed Synthesis) containing 34–36 peptides, each 18 amino acids in length and overlapping by 11 residues. One peptide pool encompassed the *SGCB* transgene (Genemed Synthesis) containing 44 peptides, each 18 amino acids in length and overlapping by 11 residues. Peptides were added directly to wells at a final concentration of 40 μg ml^−1^. Following the addition of PBMCs and peptides, the plate was incubated at 37 °C in 5–7% CO_2_ and 100% humidity for 36–48 h and then developed according to the manufacturer’s protocol. Interferon-γ spot formation was counted using Immunospot software (S6 Universal, Cellular Technology Ltd).

### ELISA

Serum samples were diluted from 1:25 to 1:26,214,400 in blocking solution (5% nonfat dry milk, 1% normal goat serum (Invitrogen) in PBS^−/−^) and added to wells for 1 h at room temperature. Sera from previously screened positive and negative human samples were used for assay controls. Goat anti-human HRP IgG-FC conjugated (Bethyl Laboratories) was used as the secondary antibody, at 1:10,000 dilution for 30 min at room temperature, followed by exposure with Ultra TMB-ELISA solution (Thermo Fisher Scientific). Absorbance of wells at optical density 450 nm was measured and used to calculate the titer of positive reaction; the lower limit of detection is 1:25.

### Immunoblot

Immunoblot was performed according to methods adapted from Pozsgai et al.^20^. SGCB levels of treatment-blinded samples were calculated from a five-point standard curve ranging from 125 to either 2,000 pg (Cohort 1, Day 60) or 3,000 pg (Cohort 1, Year 2; Cohort 2, Day 60 and Year 2) and were loaded in the same gel as the normal control pool and samples.

### qPCR

TaqMan qPCR was performed to quantify the number of vg present in targeted muscle biopsies, as previously described^26^. Biodistribution analysis was performed on tissue samples collected from three vector-dosed *mdx* animals per dose level. Tissues were harvested at necropsy, and vector-specific primer probe sets specific for sequences of the MHCK7 promoter were utilized. A vector-specific primer probe set was used to amplify a sequence of the intronic region directly downstream from the MHCK7 promoter that is unique and located within the SRP-9003 cassette. Copy number is reported as vg number per nucleus.

### ddPCR

The ddPCR assay was used to quantify vector genome copies from human muscle tissue biopsies. The human *Myogenin* gene was used as a reference to report vg per diploid human genome (also referred to as vectors per nucleus). The assay was performed using ddPCR Supermix for Probes (No dUTP) (Bio-Rad Laboratories) on a QX200 ddPCR System (Bio-Rad Laboratories) according to the manufacturer’s instructions, with a thermocycler ramp rate of 2 °C s^−1^; 95 °C for 10 min; 40 cycles of (94 °C for 30 s; 60 °C for 1 min); 98 °C for 10 min; 4 °C hold. Primers and probes (Table 1) were synthesized from Integrated DNA Technologies. Data were analyzed using QuantaSoft-Regulatory Edition v.1.7.4 (Bio-Rad Laboratories). Primers and probes used for the ddPCR assay were: vg forward primer 5′ GCAACAGACCTTTCATGGGCAAACC 3′; vg reverse primer 5′ TATAACCAGGCATCTCGGGTGTCCC 3′; human genome forward primer 5′ CTCATTGGAACTCCAGCAGGCCT 3′; human genome reverse primer 5′ GCTCTCACAGGACACCAGCTTAAAGG 3′; vg probe 5′ /56-FAM/CCCTGCTGT/ZEN/CTAGCATGCCCCACTACG/3IABkFQ/ 3′; and human genome probe 5′ /5HEX/AGCGACCTT/ZEN/CACTGGGCCACAAAATCTG/3IABkFQ/ 3′.

### Immunofluorescence

Immunostaining was conducted according to Good Clinical Laboratory Practice using qualified equipment and validated methods and software. Standard operating procedures and associated forms were utilized for related laboratory activities.

Frozen muscle samples mounted on wooden blocks were cut using a Thermo Scientific HM525NX Cryostat at 10-µm thickness. Frozen sections were mounted onto slides (Fisherbrand Superfrost Plus, catalog no. 12-550-15) and stored at −40 °C until staining. Slides were warmed to ambient temperature and fixed briefly with cold acetone (Fisher Scientific, catalog no. A18-4) and allowed to dry in air. Once dry, a hydrophobic boundary was created around frozen sections using a Pap Pen (BioGenex, catalog no. XT001PP). A no-primary slide for each antibody was stained in parallel to assess nonspecific binding of primary antibodies. A 10% goat serum blocking buffer was prepared using 1× TBS (Fisher Scientific, catalog no. BP24721) and goat serum (Gibco, catalog no. 16210064). A 1% goat serum wash solution was prepared (1× TBS, goat serum).

All steps of the staining procedure were conducted in a humidity chamber with a lid. Slides were incubated in 10% blocking buffer for 20 min, after which excess blocking buffer was removed from slides.

Primary antibody SGCD (mouse monoclonal, Leica Biosystems, catalog no. NCL-d-SARC) was diluted in 1× TBS and prepared at a dilution of 1:25. Slides were incubated for 1 h then 1× TBS was applied to the no-primary slide in place of diluted primary antibody.

Primary antibodies SGCB (mouse monoclonal, Leica Biosystems, catalog no. NCL-L-b-SARC) and SGCA (mouse monoclonal, Leica Biosystems, catalog no. NCL-L-a-SARC) were each diluted in 1× TBS and prepared at a dilution of 1:50. Slides were incubated for 1 h then 1× TBS was applied to the no-primary slide in place of diluted primary antibody.

Primary antibody SGCG (mouse monoclonal, Leica Biosystems, catalog no. NCL-g-SARC) was diluted in 1× TBS and prepared at a dilution of 1:100. Slides were incubated for 1 h then 1× TBS was applied to the no-primary slide in place of diluted primary antibody.

Excess primary antibody and 1× TBS were removed from slides after 1 h. Sections were washed three times with 1% goat serum wash solution, each for 5 min. Excess wash solution was removed from slides between each wash and after the final wash. Secondary antibody Alexa Fluor 594 (goat anti-mouse IgG Highly Cross-Adsorbed Secondary Antibody, Invitrogen, catalog no. A11032) was diluted 1:300 in 1× TBS. Slides were incubated for 1 h, and excess secondary antibody removed from slides after one further hour. Sections were washed three times with 1% goat serum wash solution, each for 5 min. Excess wash solution was removed from slides between each wash and after the final wash. Slides were then coverslipped using Vectashield antifade mounting medium (Vector Labs, catalog no. H-100010) and glass coverslips (Gold Seal Coverslips, Electron Microscopy Sciences, catalog no. 6376910) and stored at −40 °C.

Depending on the time of patient enrollment and biopsy timepoints, images were captured using either an Axioskop microscope with AxioVision Rel software (Zeiss) or an ECLIPSE N*i*-U microscope (Nikon) fitted with a PROKYON camera equipped with GRYPHAX software (Jenoptik). All fresh-frozen muscle samples (four blocks per patient per timepoint) included four nonoverlapping images of immunofluorescence-labeled tissue sections, each at ×20 objective. Positive and negative control tissues per batch run were used to set an optimum exposure time. All images were captured in an identical manner and with the same exposure time for each batch session. Proper storage conditions of the tissue samples and stained slides protected stability for optimal fluorophore signal intensity without reduction of expression. Images were saved for analysis in .tiff format using eight bits per channel for fluorochrome images captured for image analysis. For imaging analysis, four ×20 images per tissue block were taken, with four per timepoint (that is, 16 images per patient per timepoint).

### BIOQUANT image analysis

Images (×20) were measured using BIOQUANT software v.2019. To determine the upper and lower thresholds for analysis, the normal and negative control images were used by selecting the stained membrane, including the various shades of intensity within the run. Threshold settings for the dataset were obtained from two trained technical analysts independently. Threshold values obtained for red, green and blue channels were averaged, with the average value for each channel taken as the consensus threshold for that dataset. A sequence of images was analyzed by selecting the field measurement tool. Values for Fluorescence Quantitation (D6) and Red Fluorescence Intensity (D3) BIOQUANT measurement algorithms were recorded. An average value was calculated for expression percentage of normal controls for the dataset, which was normalized as 100%.

### Percentage positive fiber analysis

Images (×20) were assessed for the relative number of positive fibers compared with total fibers based on visual examination of digital images. SGCB-positive fiber established criteria were defined as positive if the membrane contained approximately 30% or greater additive staining of the fiber perimeter (membrane) above the low-intensity levels of the run, including controls. The total percentage of the fiber perimeter did not need to be continuous and was subjectively determined by consciously combining all segments of perimeter positivity to determine a total perimeter intensity percentage. Fibers counted were defined by the structural appearance of their cross-section. To facilitate scoring, NIH ImageJ with the Cell Counter plugin was used to count total fibers. By convention, a ʻType #ʼ and color was selected to score/mark positive fibers. The Cell Counter tracked counts as fibers were selected. Positive fibers were scored based on the original image exposure; there was no adjustment to the brightness or contrast of any image during the positive-image scoring process. Once positive fiber selections were completed, the brightness and contrast of the image were adjusted to visualize negative fibers more easily. No additional positive fibers were scored once brightness/contrast was adjusted. The remaining fibers were scored as negative using a different Type # and color for the score/mark. Once scoring was complete, ʻExport Imageʼ was selected from the Cell Counter workspace and this image was saved separately as an annotated .jpeg image. Total fiber counts were determined after all fibers were counted.

### Functional assessments

Functional efficacy was assessed using NSAD, a 29-item activity-level scale for measurement of motor performance, with a score range of 0–54 (higher scores indicate a higher level of function)^35–38^. Other functional outcomes included time to rise, four-stair climb, 100-m test, 10-m test and hand-held dynamometry for knee extensors and flexors and elbow flexors.

### NCH natural history cohort

The NCH natural history study is a prospective, observational, longitudinal cohort study assessing the natural history of LGMD2A and LGMD2E (ClinicalTrials.gov identifier: NCT03488784)^37^. To select individuals in the untreated NCH natural history cohort for comparison with treated patients in the SRP-9003-101 trial, the predefined inclusion criteria for SRP-9003-101 were also applied to the natural history dataset, as follows:

1. LGMD2E/R4 ambulatory subjects only; for the natural history cohort, this was defined as those with nonmissing 10-m test value
2. Baseline age 4–15 years, inclusive
3. 100-m test result: ≥40% of that predicted for age-, height-, gender- and weight-matched healthy controls

Supplementary Fig. 2 shows the derivation of the natural history comparator group. From an overall dataset of 35 subjects, five control subjects who met these criteria were identified. The matched patients were originally consented to NCT03488784 from November 2016 to August 2019.

### Statistics and reproducibility

Sample size was based on enrollment feasibility; there was no formal determination based on statistical considerations. Analyses were primarily descriptive in nature and no formal statistical tests were performed. Descriptive statistics are presented for all endpoints and included number of subjects (*n*), mean, s.d., minimum and maximum for continuous variables and number and percentage for categorical variables. Baseline and demographic characteristics, safety, biopsy and functional data were summarized using descriptive statistics. No sex- or gender-based analysis was performed owing to the small sample size. SAS v.9.4 (SAS Institute Inc.) was used for demographics and safety data, and Prism v.5 (GraphPad Software) for biopsy data. For the NCH natural history cohort, baseline was defined as the first timepoint where both the 100-m test and NSAD were nonmissing.

For each patient and timepoint, four biopsies were collected. Each biopsy was analyzed with two technical replicates using a validated immunoblot method. The immunoblot experiment was not repeated after obtaining valid results from two technical replicates. The average coefficient of variation of technical replicates was 10.17%. During validation of the immunoblot method, the following parameters were assessed and deemed suitable for the analysis of study samples: accuracy, precision, specificity, sensitivity, linearity and measurement of range and robustness. Validation was performed based on Food and Drug Administration guidelines.

### Reporting summary

Further information on research design is available in the Nature Portfolio Reporting Summary linked to this article.

## Online content

Any methods, additional references, Nature Portfolio reporting summaries, source data, extended data, supplementary information, acknowledgements, peer review information; details of author contributions and competing interests; and statements of data and code availability are available at 10.1038/s41591-023-02730-9.

### Supplementary information

Supplementary Information Supplementary Tables 1 and 2 and Figs. 1–3.

Reporting Summary

### Source data

Source Data Extended Data Fig. 2 Unprocessed immunoblots used in Extended Data Fig. 2.

## Figures and Tables

**Extended Data Table 1:**
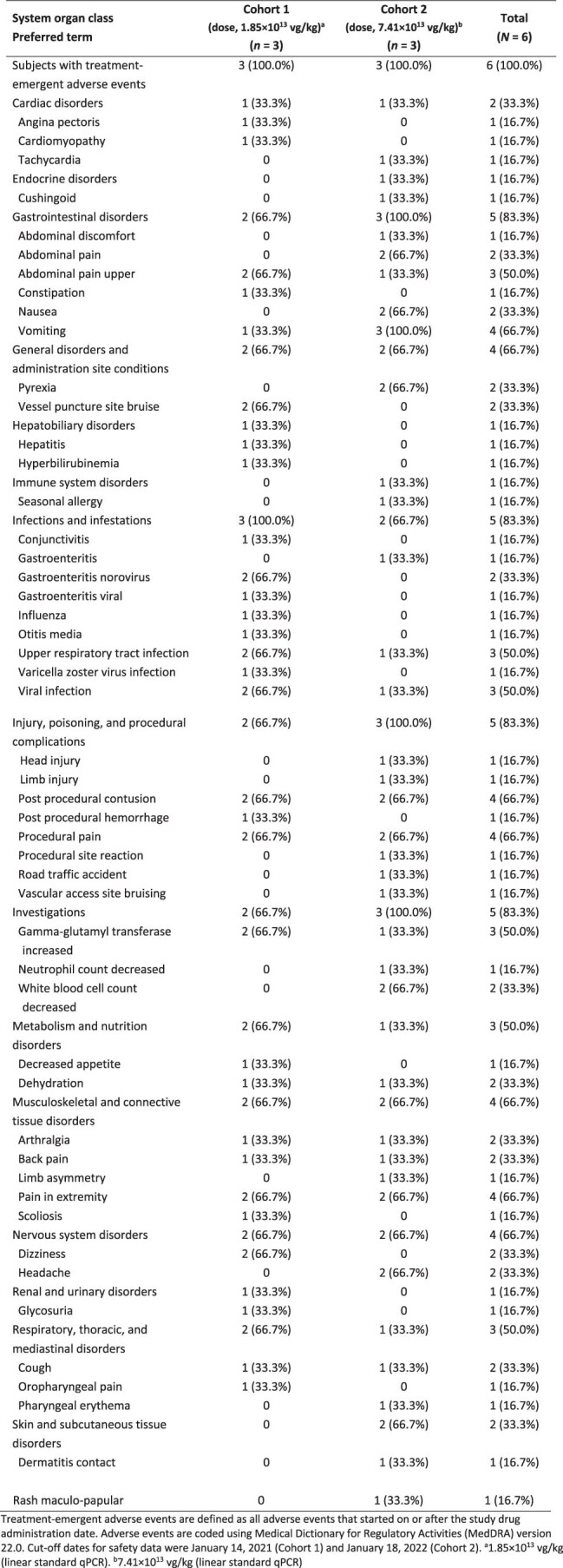
Primary outcome of treatment-emergent adverse events by system organ class and preferred term at 2 years

**Extended Data Table 2:**
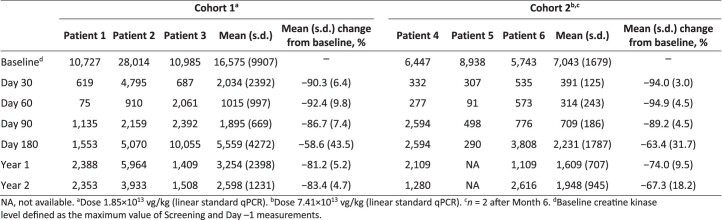
Exploratory outcome of serum creatine kinase levels (U/L) throughout the study

**Extended Data Table 3:**
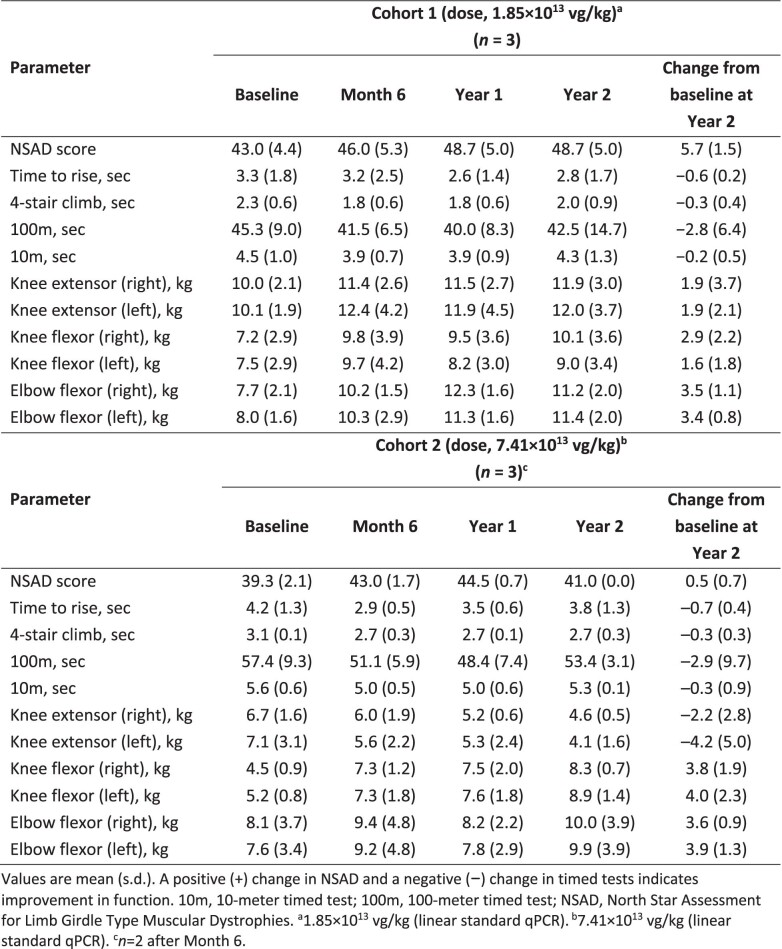
Exploratory outcomes of additional functional outcome measures assessed throughout the study: Cohort 1 and Cohort 2

**Extended Data Table 4:**
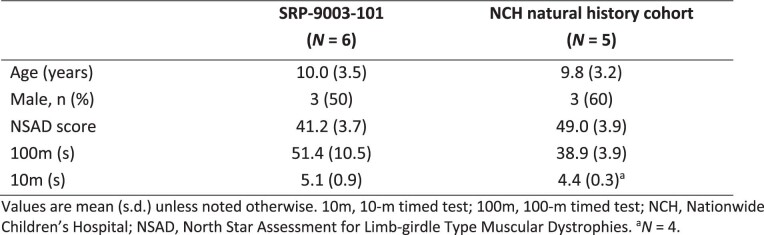
Post hoc exploratory baseline comparison of bidridistrogene xeboparvovec–treated patients versus natural history cohort

**Extended Data Table 5:**
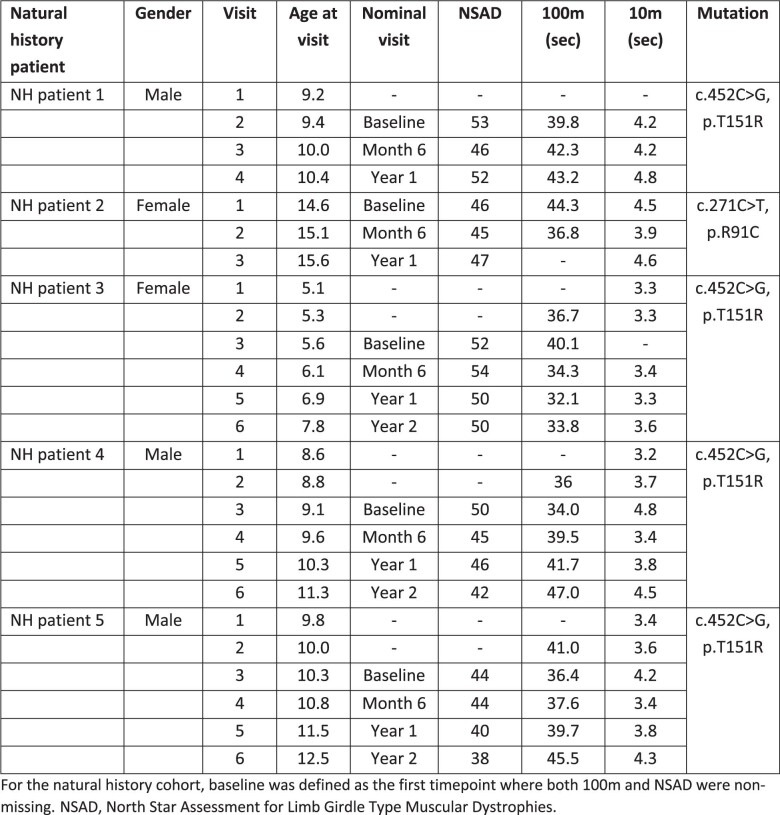
Exploratory functional outcome measures: individual patient data for the matched natural history cohort

**Extended Data Table 6:**
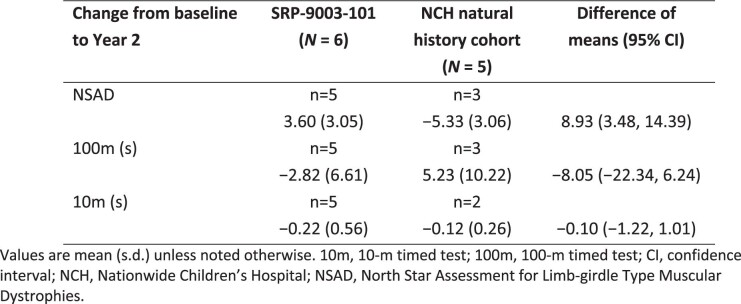
Post hoc exploratory analysis – descriptive summary of functional outcomes between the treated cohort and the matched natural history cohort

## Data Availability

Individual data for all patients are presented in the paper but, for any further information, qualified researchers may reach out to Sarepta Therapeutics, Inc. by contacting medinfo@sarepta.com. Data requests will be fulfilled within 90 days. Because the study is ongoing, access to the data is limited to those that support the findings of this study. Source data are provided with this paper.
