# Supplementary material for: Gene therapy with bidridistrogene xeboparvovec for limb-girdle muscular dystrophy type 2E/R4: phase 1/2 trial results
Source: Nat Med. 2024 Jan 4;30(1):199–206. doi: 10.1038/s41591-023-02730-9 (PMC10803256; doi:10.1038/s41591-023-02730-9)

nature medicine

Article

https://doi.org/10.1038/s41591-023-02730-9

Gene therapy with bidridistrogene xeboparvovec for limb-girdle muscular dystrophy type 2E/R4: phase 1/2 trial results

In the format provided by the
authors and unedited

SUPPLEMENTARY NOTE
Vector sequence
Supplementary Table 1 | Primary and exploratory outcomes: Individual patient data for β-sarcoglycan and sarcoglycan complex expression by immunofluorescence percent positive fibers by muscle.

Supplementary Table 2 | Primary and exploratory outcomes: Individual patient data for β-sarcoglycan and sarcoglycan complex expression by immunofluorescence fiber intensity by muscle.

Supplementary Figure 1. SRP-9003: Self-complementary AAV vector .

Supplementary Fig. 2 | Derivation of the natural history comparator group

Supplementary Fig. 3 | Raw uncut images of all western blots for all patients from both Cohort 1 and Cohort 2 for baseline, D60 and Year 2 stained for SGCB and actinin loading control. Patient identifiers were blinded, as were test operators. Samples were run in duplicate in multiple gels. Each blot included the normal control (NC) pool (representing wild-type control), and a 5-point standard curve utilizing increasing amounts of recombinant protein (labeled 'reSGCB')

Vector sequence:

1 CTGCGCGCTC GCTCGCTCAC TGAGGCCGCC CGGGCAAAGC CCGGGCGTCG

51 GGCGACCTTT GGTCGCCCGG CCTCAGTGAG CGAGCGAGCG CGCAGAGAGG

101 GAGTGGCCAA CTCCATCACT AGGGGTTCCT TGTAGTTAAT GATTAACCCG

151 CCATGCTACT TATCTACGTA GCCATGCTCT AGAGGCGCGC CCCTGCAGGA

201 CACACAAAAA ACCAACACAC AGATCTAATG AAAATAAAGA TCTTTTATTG

251 CGGCCAAGCT TTTAATGAGT ATTCCCACAA GGGTTATCGC TAATCTGACA

301 TCCCATATTC TGGCTGGTGA CCTGCACTTT AAACAGGGTG CCATCGGCAC

351 ACATGCACAG CTTATACCTG ACCCAATCTC CGGACCCCAG CTGGTCTCCA

401 CTAGAGGAGC TGGGCAGTCT GGTAGTGGAG ACCATCACGC TCCCATTCAG

451 AATGATGCTG TTTTCTGCTT TCAGCTCCAT GTTGCCTCCC ATGTGAAATT

501 CAATTGTCTT GCCCATGATG AACACGCCCT CATTTCCCCG GACAATAGCT

551 CGTCCATCCA CCTTGATATT CAGGTCTGAT GTAGCGTTAC TGGTGATTCT

601 CTCAGTGCTG GCTTTCTGGA CGTTCAGAGA CTTCACCCCG GAAGGCAGAT

651 GAAATTCGTG TGTCTCATAG TCGGTACTGA ACAGGATATT CTGGGTCCGG

701 GGATCAAAGA ACTGCATGCC AATGTCGCTA GTGATTGATG TTTTATTGTT

751 TTCCACAGAC AGCTTTGTGG TTCCCTGCTG GAACACAATG GGCTGATTGT

801 TCCCGGTGAT CACCAGATTC TCGTTTCTCC GCCCGCCGAC AGTAGATTTG

851 TACAGTGGAT GGATGACCCC CATATCGGAC ACCTGCTTAA ATCGCAGCAG

901 GCCACTTTCG TGGAACTCCA TAGAGTCACA CCCGTTTGGG CCAATGCGGA

951 TGACAGCCCA AATCACCAGA GTAATGATCA GATTAATCAC GGCCAGGATA

1001 AACAGCAGAA TGATGACGCA GATTGCCAGG TTTCCTTTGC GCCCCCTCAG

1051 GCCTGTCTTA TGCAGGCGAT CTTCGTCAAT AGGGATGTAG CCGGCTTTGA

1101 AATTGCTGTT GTGCTCCTTA TTCACTGATC TCCTCTCGAC GGCTTTTTCT

1151 CTCATTGATT TTTTCACTGG TCCATTGCTT GACTGCTGCT CGGCGGCTGC

1201 GGCGGCTGCT GCTGCCATGG TGGTACCGGG TACAATTCCG CAGCTTTTAG

1251 AGCAGAAGTA ACACTTCCGT ACAGGCCTAG AAGTAAAGGC AACATCCACT

1301 GAGGAGCAGT TCTTTGATTT GCACCACCAC CGGATCCGGG ACCTGAAATA

1351 AAAGACAAAA AGACTAAACT TACCTGGGCG CGCCGCTGGC TGCTCCTGAG

1401 TGTCTGTCTG TGCTGTGGAG GTGGTGGTAG AATGAGGGCA GCCCCTGTGC

1451 CCCTGGGTTA TATAGAGGAG CCTACAGGGT GTGACTAGCC AGGAGGGGCT

1501 GTCCCCAGGG AGGGGCCCCT GAGAGCAGAT GAGCTTTCAG CTCGTTGCCC

1551 GGGCACCGTG CCCACCCCGG ACCCAGGCGT GCAGCTTGCC CAGCCCCATG

1601 GCCTTGTATG GGCTGCCCCA AGGGCTGACT TGCTCACTGG TTCCTAAACT

1651 AAGTGCTGAG TCTAGCTGGC GGGGGACAGC TGGCCCTTCG CCGGGAACAT

1701 GGAACAGTAA TACTTTGGGA GTCCCAGGCA CGTATAAGCC CTGGCCCCCA

1751 AGCCTGTTAC AGCCTGCCCT CAGTCCCCCA CAGCCTTGTT CGAAGATCTT

1801 CGCATGCAGG GGATCCACCA GGGACAGGGT TATTTTTAGA GGCAGCAGGT

1851 GTTGGGGGGG GGGGGGCAGC CACATGTCTG GGTTAATTAT AACCAGGCAT

1901 CTCGGGTGTC CCCAGGCCTT GCCTCCTTAC ATGGGCAGCC TAGACCCGTA

1951 GTGGGGCATG CTAGACAGCA GGGCCCCAAG GTTTGCCCAT GAAAGGTCTG

2001 TTGCCCTCGC CCCTCTGGCT CCATGGCCTT TTTTTAGTCC TTGGGCACAT

2051 TCCTCCTCCC CAAAGGGCCG ATGGGCAGAT AGAGGAGAGA CAGGAGCGTC

2101 TCACACCACC TCCCCTACCC AGGCCCTTAC CTCAGTTATT TTTAATCTGA

2151 AGGGTCTAGC TTAGACATGC AAGCTTGCGG CCGCCAATTG GTTAACCCCA

2201 CTCCCTCTCT GCGCGCTCGC TCGCTCACTG AGGCCGCCCG GGCAAAGCCC

2251 GGGCGTCGGG CGACCTTTGG TCGCCCGGCC TCAGTGAGCG AGCGAGCGCG

2301 CAGAGAGGGA GTGGGGTTAA CCAATTGGCG GCCGCAAGCT TGCATGTCTA

2351 AGCTAGACCC TTCAGATTAA AAATAACTGA GGTAAGGGCC TGGGTAGGGG

2401 AGGTGGTGTG AGACGCTCCT GTCTCTCCTC TATCTGCCCA TCGGCCCTTT

2451 GGGGAGGAGG AATGTGCCCA AGGACTAAAA AAAGGCCATG GAGCCAGAGG

2501 GGCGAGGGCA ACAGACCTTT CATGGGCAAA CCTTGGGGCC CTGCTGTCTA

2551 GCATGCCCCA CTACGGGTCT AGGCTGCCCA TGTAAGGAGG CAAGGCCTGG

2601 GGACACCCGA GATGCCTGGT TATAATTAAC CCAGACATGT GGCTGCCCCC

2651 CCCCCCCCAA CACCTGCTGC CTCTAAAAAT AACCCTGTCC CTGGTGGATC

2701 CCCTGCATGC GAAGATCTTC GAACAAGGCT GTGGGGGACT GAGGGCAGGC

2751 TGTAACAGGC TTGGGGGCCA GGGCTTATAC GTGCCTGGGA CTCCCAAAGT

2801 ATTACTGTTC CATGTTCCCG GCGAAGGGCC AGCTGTCCCC CGCCAGCTAG

2851 ACTCAGCACT TAGTTTAGGA ACCAGTGAGC AAGTCAGCCC TTGGGGCAGC

2901 CCATACAAGG CCATGGGGCT GGGCAAGCTG CACGCCTGGG TCCGGGGTGG

2951 GCACGGTGCC CGGGCAACGA GCTGAAAGCT CATCTGCTCT CAGGGGCCCC

3001 TCCCTGGGGA CAGCCCCTCC TGGCTAGTCA CACCCTGTAG GCTCCTCTAT

3051 ATAACCCAGG GGCACAGGGG CTGCCCTCAT TCTACCACCA CCTCCACAGC

3101 ACAGACAGAC ACTCAGGAGC AGCCAGCGGC GCGCCCAGGT AAGTTTAGTC

3151 TTTTTGTCTT TTATTTCAGG TCCCGGATCC GGTGGTGGTG CAAATCAAAG

3201 AACTGCTCCT CAGTGGATGT TGCCTTTACT TCTAGGCCTG TACGGAAGTG

3251 TTACTTCTGC TCTAAAAGCT GCGGAATTGT ACCCGGTACC ACCATGGCAG

3301 CAGCAGCCGC CGCAGCCGCC GAGCAGCAGT CAAGCAATGG ACCAGTGAAA

3351 AAATCAATGA GAGAAAAAGC CGTCGAGAGG AGATCAGTGA ATAAGGAGCA

3401 CAACAGCAAT TTCAAAGCCG GCTACATCCC TATTGACGAA GATCGCCTGC

3451 ATAAGACAGG CCTGAGGGGG CGCAAAGGAA ACCTGGCAAT CTGCGTCATC

3501 ATTCTGCTGT TTATCCTGGC CGTGATTAAT CTGATCATTA CTCTGGTGAT

3551 TTGGGCTGTC ATCCGCATTG GCCCAAACGG GTGTGACTCT ATGGAGTTCC

3601 ACGAAAGTGG CCTGCTGCGA TTTAAGCAGG TGTCCGATAT GGGGGTCATC

3651 CATCCACTGT ACAAATCTAC TGTCGGCGGG CGGAGAAACG AGAATCTGGT

3701 GATCACCGGG AACAATCAGC CCATTGTGTT CCAGCAGGGA ACCACAAAGC

3751 TGTCTGTGGA AAACAATAAA ACATCAATCA CTAGCGACAT TGGCATGCAG

3801 TTCTTTGATC CCCGGACCCA GAATATCCTG TTCAGTACCG ACTATGAGAC

3851 ACACGAATTT CATCTGCCTT CCGGGGTGAA GTCTCTGAAC GTCCAGAAAG

3901 CCAGCACTGA GAGAATCACC AGTAACGCTA CATCAGACCT GAATATCAAG

3951 GTGGATGGAC GAGCTATTGT CCGGGGAAAT GAGGGCGTGT TCATCATGGG

4001 CAAGACAATT GAATTTCACA TGGGAGGCAA CATGGAGCTG AAAGCAGAAA

4051 ACAGCATCAT TCTGAATGGG AGCGTGATGG TCTCCACTAC CAGACTGCCC

4101 AGCTCCTCTA GTGGAGACCA GCTGGGGTCC GGAGATTGGG TCAGGTATAA

4151 GCTGTGCATG TGTGCCGATG GCACCCTGTT TAAAGTGCAG GTCACCAGCC

4201 AGAATATGGG ATGTCAGATT AGCGATAACC CTTGTGGGAA TACTCATTAA

4251 AAGCTTGGCC GCAATAAAAG ATCTTTATTT TCATTAGATC TGTGTGTTGG

4301 TTTTTTGTGT GTCCTGCAGG GGCGCGCCTC TAGAGCATGG CTACGTAGAT

4351 AAGTAGCATG GCGGGTTAAT CATTAACTAC AAGGAACCCC TAGTGATGGA

4401 GTTGGCCACT CCCTCTCTGC GCGCTCGCTC GCTCACTGAG GCCGGGCGAC

4451 CAAAGGTCGC CCGACGCCCG GGCTTTGCCC GGGCGGCCTC AGTGAGCGAG

4501 CGAGCGCGCA G

Supplementary Table 1 | Primary and exploratory outcomes: Individual patient data for β-sarcoglycan and sarcoglycan complex expression by immunofluorescence percent positive fibers by muscle.

Patient (cohort)	Parameter	Visit	Tibialis anterior	Biceps	Total
Patient 1 (cohort 1)	SGCB	Baseline	2.605	14.435	8.520
		Day 60	54.610	70.445	62.528
		Year 2	32.385	12.120	22.253
	SGCA	Baseline	4.660	7.595	6.128
		Day 60	43.675	60.690	52.183
		Year 2	18.610	2.385	10.498
	SGCD	Baseline	9.030	5.885	7.458
		Day 60	49.830	58.025	53.928
		Year 2	29.405	10.890	20.148
	SGCG	Baseline	0.000	0.325	0.163
		Day 60	12.725	33.030	22.878
		Year 2	32.205	6.690	19.448
Patient 2 (cohort 1)	SGCB	Baseline	8.030	0.675	4.353
		Day 60	71.050	26.760	48.905
		Year 2	61.060	56.170	58.615
	SGCA	Baseline	2.100	0.615	1.358
		Day 60	68.820	31.370	50.095
		Year 2	43.995	44.260	44.128
	SGCD	Baseline	13.050	6.460	9.755
		Day 60	53.910	43.590	48.750
		Year 2	49.060	40.970	45.015
	SGCG	Baseline	0.000	0.370	0.185
		Day 60	60.500	26.155	43.328
		Year 2	58.525	45.920	52.223
Patient 3 (cohort 1)	SGCB	Baseline	20.690	6.710	13.700
		Day 60	50.415	32.855	41.635
		Year 2	56.155	63.190	59.673

	SGCA	Baseline	26.250	19.035	22.643
		Day 60	55.700	27.250	41.475
		Year 2	37.295	50.120	43.708
	SGCD	Baseline	27.855	15.840	21.848
		Day 60	49.880	38.545	44.213
		Year 2	41.095	58.060	49.578
	SGCG	Baseline	19.425	0.880	10.153
		Day 60	27.035	15.160	21.098
		Year 2	51.860	60.390	56.125
Patient 4	SGCB	Baseline	18.465	7.105	12.785
(cohort 2)		Day 60	86.145	44.380	65.263
		Year 2	41.735	54.010	47.873
	SGCA	Baseline	26.050	26.645	26.348
		Day 60	76.230	48.215	62.223
		Year 2	59.035	73.970	66.503
	SGCD	Baseline	25.355	18.035	21.695
		Day 60	84.050	44.735	64.393
		Year 2	62.985	71.795	67.390
	SGCG	Baseline	11.715	14.770	13.243
		Day 60	83.560	34.010	58.785
		Year 2	64.680	67.530	66.105
Patient 5	SGCB	Baseline	7.130	2.965	5.048
(cohort 2)		Day 60	75.595	78.315	76.955
	SGCA	Baseline	25.025	22.245	23.635
		Day 60	76.125	73.645	74.885
	SGCD	Baseline	11.310	1.950	6.630
		Day 60	72.435	76.920	74.678
	SGCG	Baseline	3.925	0.000	1.963
		Day 60	82.115	69.575	75.845
Patient 6	SGCB	Baseline	0.000	0.000	0.000
(cohort 2)		Day 60	84.015	65.550	74.783
		Year 2	81.165	75.545	78.355
	SGCA	Baseline	15.310	17.565	16.438
		Day 60	86.870	78.145	82.508
		Year 2	85.280	75.910	80.595
	SGCD	Baseline	5.345	3.070	4.208
		Day 60	85.185	77.550	81.368
		Year 2	85.220	73.415	79.318
	SGCG	Baseline	0.000	0.135	0.068

		Day 60	92.175	75.090	83.633
		Year 2	85.550	77.535	81.543

Patient 5 died in an accident unrelated to the study and therefore the Year 2 biopsy timepoint is missing. SGCA, α-sarcoglycan; SGCB, β-sarcoglycan; SGCD, δ-sarcoglycan; SGCG, γ-sarcoglycan.

Supplementary Table 2 | Primary and exploratory outcomes: Individual patient data for β-sarcoglycan and sarcoglycan complex expression by immunofluorescence fiber intensity by muscle.

Patient (cohort)	Parameter	Visit	Tibialis anterior	Biceps	Total
Patient 1 (cohort 1)	SGCB	Baseline	3.075	3.435	3.255
		Day 60	32.395	62.350	47.373
		Year 2	15.685	1.940	8.813
	SGCA	Baseline	6.880	9.940	8.410
		Day 60	35.920	61.930	48.925
		Year 2	13.690	4.305	8.998
	SGCD	Baseline	0.395	0.700	0.548
		Day 60	3.670	13.985	8.828
		Year 2	8.805	1.450	5.128
	SGCG	Baseline	0.075	0.030	0.053
		Day 60	0.050	4.460	2.255
		Year 2	10.875	0.580	5.728
Patient 2 (cohort 1)	SGCB	Baseline	8.720	0.765	4.743
		Day 60	94.340	19.515	56.928
		Year 2	58.190	42.830	50.510
	SGCA	Baseline	34.470	21.800	28.135
		Day 60	100.525	48.625	74.575
		Year 2	35.265	38.755	37.010
	SGCD	Baseline	4.940	1.360	3.150
		Day 60	17.540	7.880	12.710
		Year 2	27.420	28.995	28.208
	SGCG	Baseline	0.615	1.240	0.928
		Day 60	22.295	1.155	11.725
		Year 2	38.410	20.910	29.660
Patient 3 (cohort 1)	SGCB	Baseline	17.320	11.235	14.278
		Day 60	46.720	29.275	37.998
		Year 2	35.050	56.820	45.935

	SGCA	Baseline	45.015	38.285	41.650
		Day 60	55.135	38.930	47.033
		Year 2	26.460	34.640	30.550
	SGCD	Baseline	33.455	21.480	27.468
		Day 60	32.095	27.070	29.583
		Year 2	23.365	44.895	34.130
	SGCG	Baseline	38.360	32.830	35.595
		Day 60	45.810	29.045	37.428
		Year 2	22.350	27.260	24.805
Patient 4	SGCB	Baseline	13.155	8.995	11.075
(cohort 2)		Day 60	93.220	16.530	54.875
		Year 2	10.935	29.140	20.038
	SGCA	Baseline	50.040	56.065	53.053
		Day 60	105.350	61.115	83.233
		Year 2	31.300	68.370	49.835
	SGCD	Baseline	14.270	21.460	17.865
		Day 60	76.555	24.375	50.465
		Year 2	18.100	58.395	38.248
	SGCG	Baseline	13.900	7.260	10.580
		Day 60	86.215	14.960	50.588
		Year 2	25.100	55.390	40.245
Patient 5	SGCB	Baseline	3.400	1.625	2.513
(cohort 2)		Day 60	67.660	66.660	67.160
	SGCA	Baseline	51.270	30.310	40.790
		Day 60	89.845	104.225	97.035
	SGCD	Baseline	9.345	3.795	6.570
		Day 60	58.035	55.220	56.628
	SGCG	Baseline	5.200	0.140	2.670
		Day 60	74.620	50.065	62.343
Patient 6	SGCB	Baseline	0.125	0.200	0.163
(cohort 2)		Day 60	117.425	77.160	97.293
		Year 2	68.825	65.185	67.005
	SGCA	Baseline	6.285	11.115	8.700
		Day 60	122.580	92.905	107.743
		Year 2	127.900	89.100	108.500
	SGCD	Baseline	0.605	0.785	0.695
		Day 60	85.275	65.305	75.290
		Year 2	86.800	50.510	68.655
	SGCG	Baseline	0.300	0.195	0.248

	Day 60	117.025	75.170	96.098
	Year 2	89.600	68.175	78.888

Patient 5 died in an accident unrelated to the study and therefore the Year 2 biopsy timepoint is missing. SGCA, α-sarcoglycan; SGCB, β-sarcoglycan; SGCD, δ-sarcoglycan; SGCG, γ-sarcoglycan.

Supplementary Figure 1. SRP-9003: Self-complementary AAV vector .

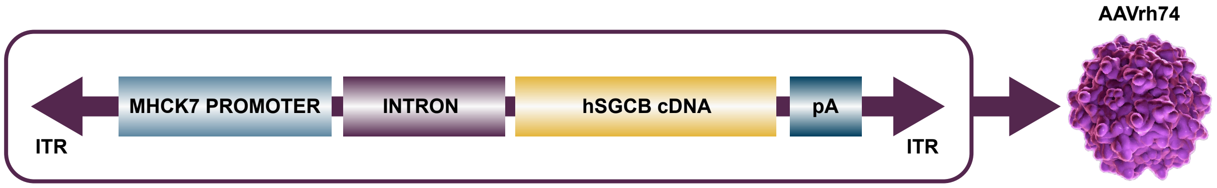

AAV, adeno-associated virus; cDNA, complementary deoxyribonucleic acid; hSGCB, human β-sarcoglycan; ITR, inverted terminal repeat; MHCK7, myosin heavy chain enhancer promoter; pA, polyadenylation.

Supplementary Fig. 2 | Derivation of the natural history comparator group

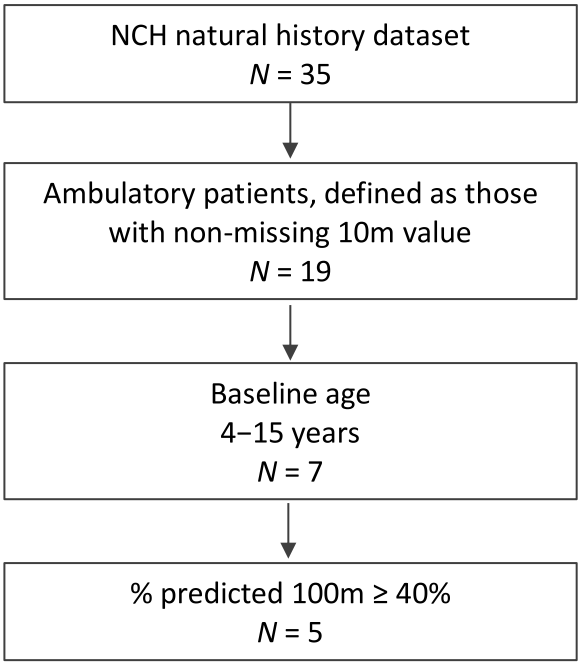

10m, 10m-meter walk/run;100m, 100-meter walk/run; NCH, Nationwide Children’s Hospital

Supplementary Fig. 3 | Raw uncut images of all western blots for all patients from both Cohort 1 and Cohort 2 for baseline, D60 and Year 2 stained for SGCB and actinin loading control. Patient identifiers were blinded, as were test operators. Samples were run in duplicate in multiple gels. Each blot included the normal control (NC) pool (representing wild-type control), and a 5-point standard curve utilizing increasing amounts of recombinant protein (labeled 'reSGCB')

A: Patient-1,2 and 3: pretreatment and day 60 SGCB staining (samples in duplicates; loading control not available)

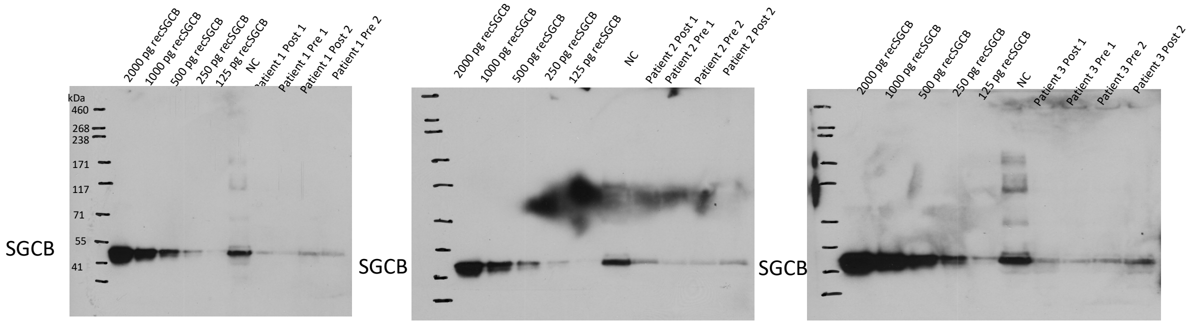

B: Patients-1, 2, and 3: Year-2 SGCB staining (samples in duplicates)

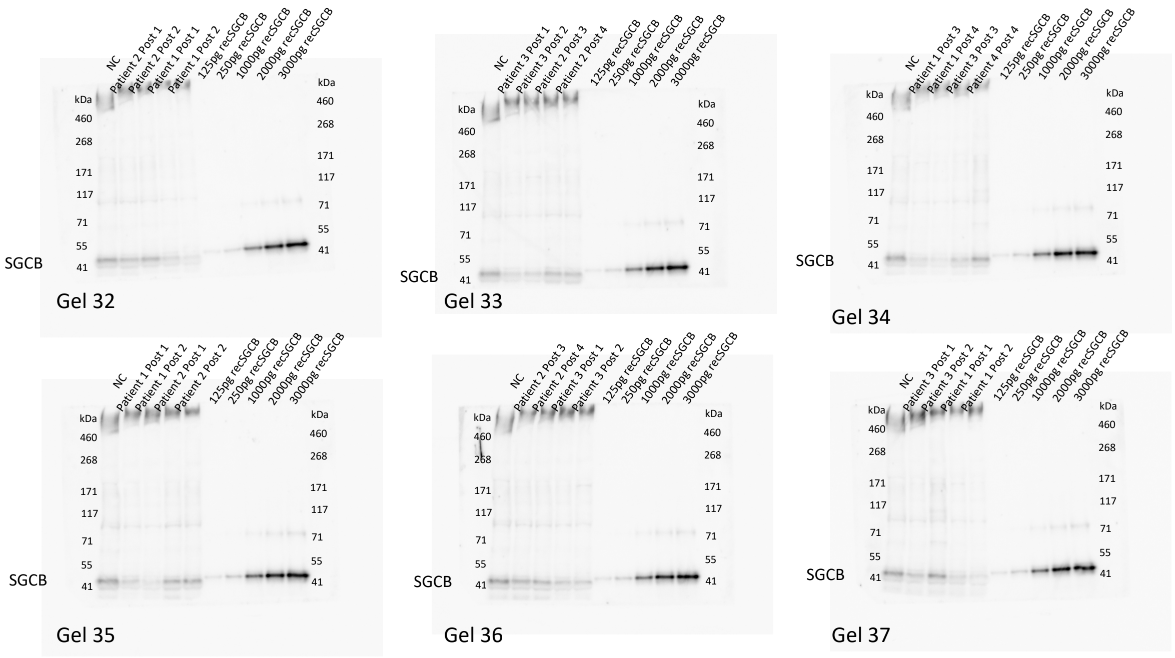

C: Patients-1, 2, and 3: Year-2 actinin staining (samples in duplicates)

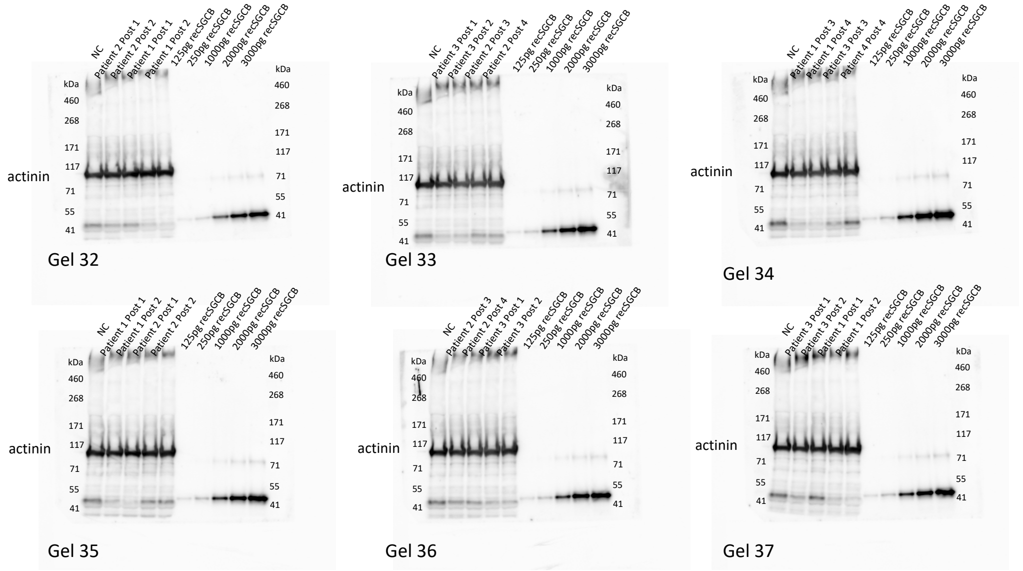

D: Patients- 4 and 5: pretreatment and Day 60 SGCB staining (samples in duplicates)

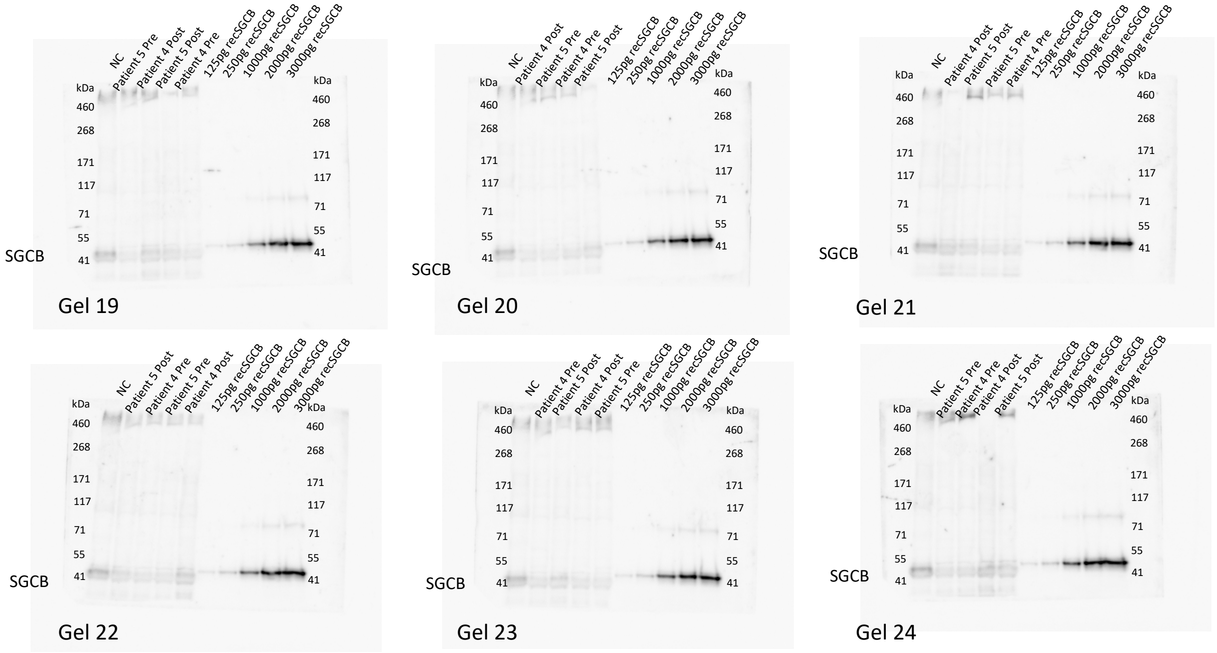

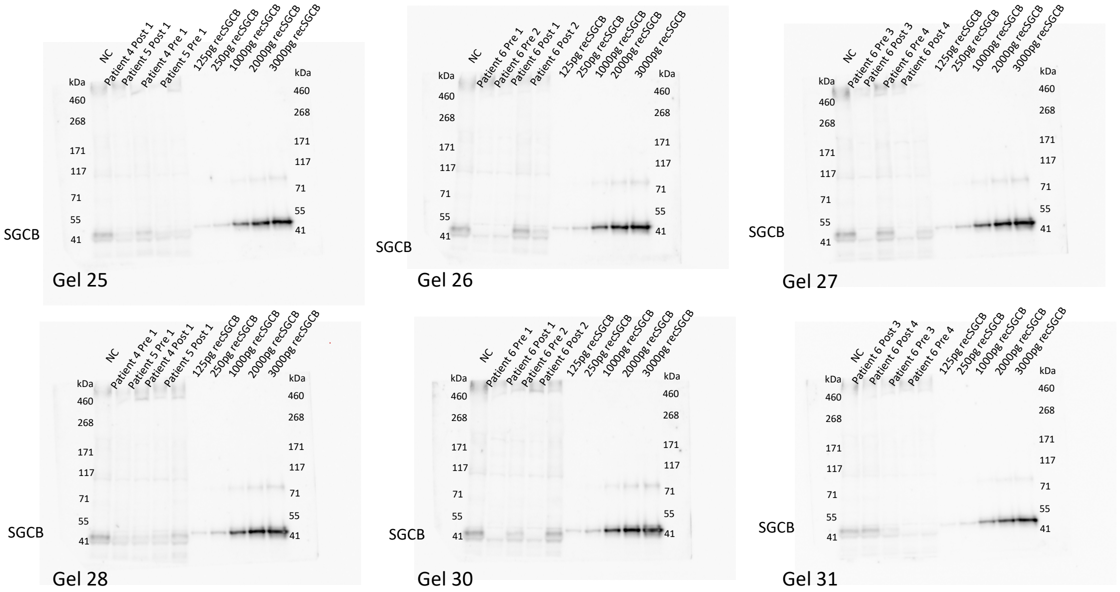

E: Patients- 4 and 5: pretreatment and Day 60 actinin staining (samples in duplicates)

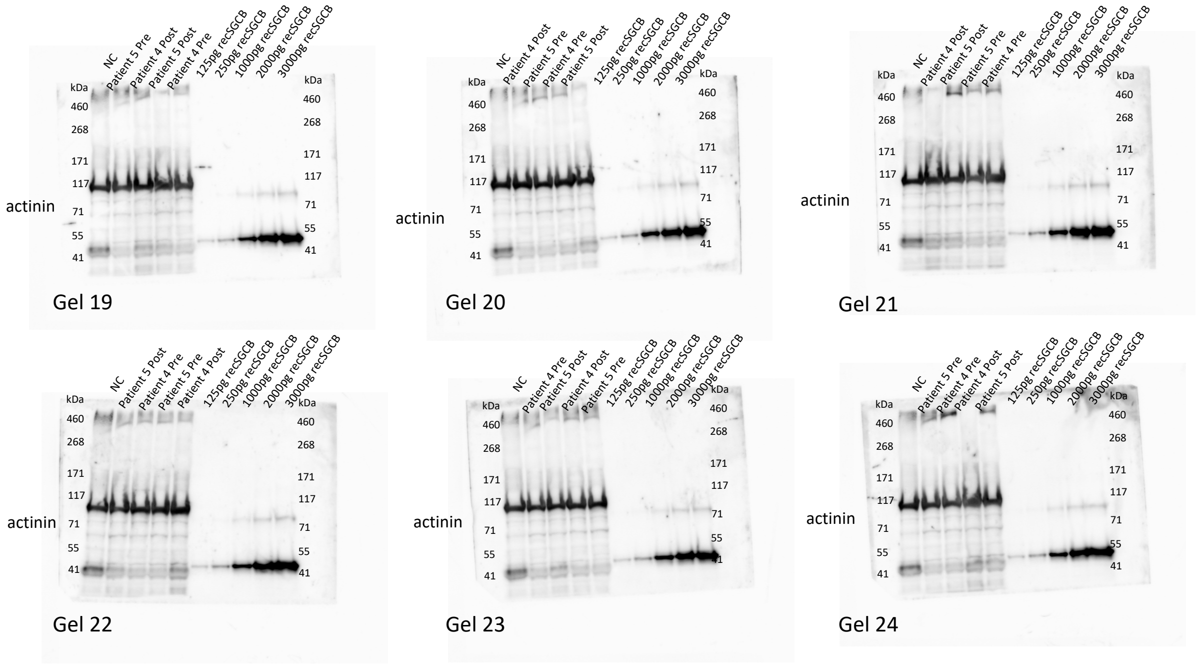

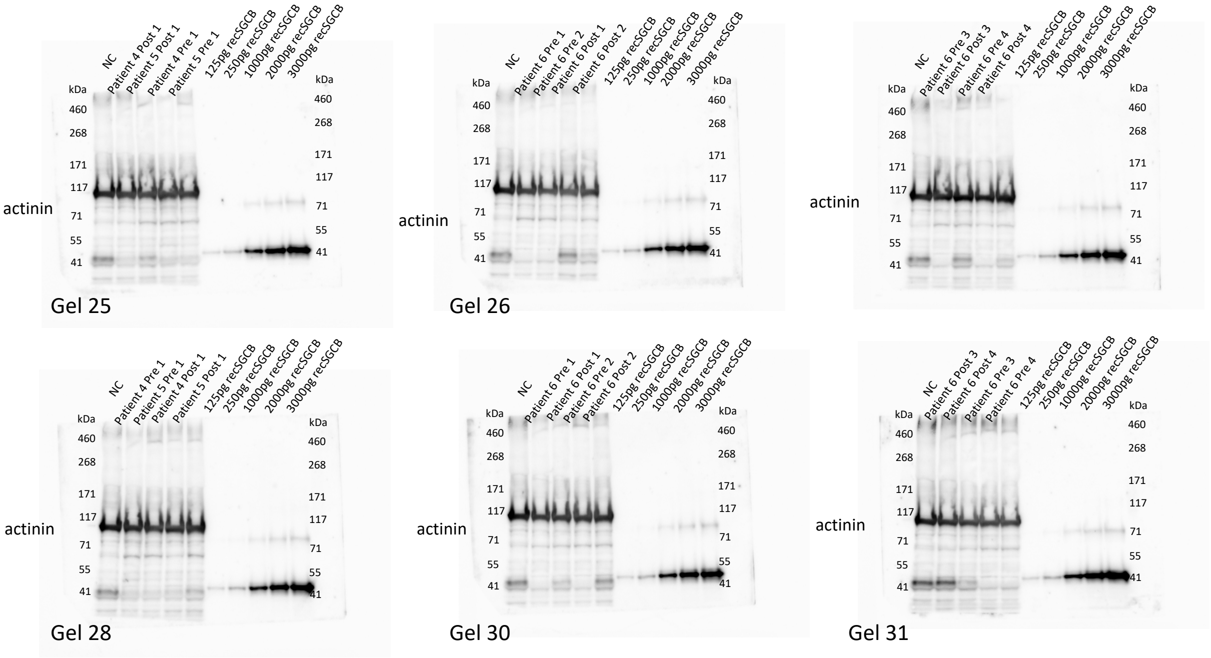

F: Patients- 4 and 5: Year-2 SGCB staining (samples in duplicates)

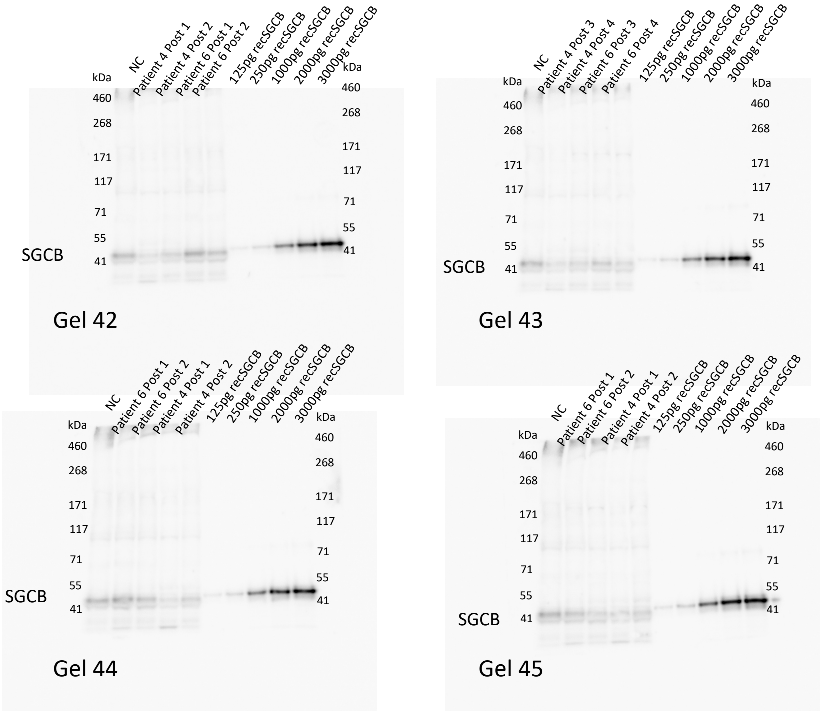

G: Patients- 4 and 5: Year-2 actinin staining (samples in duplicates)

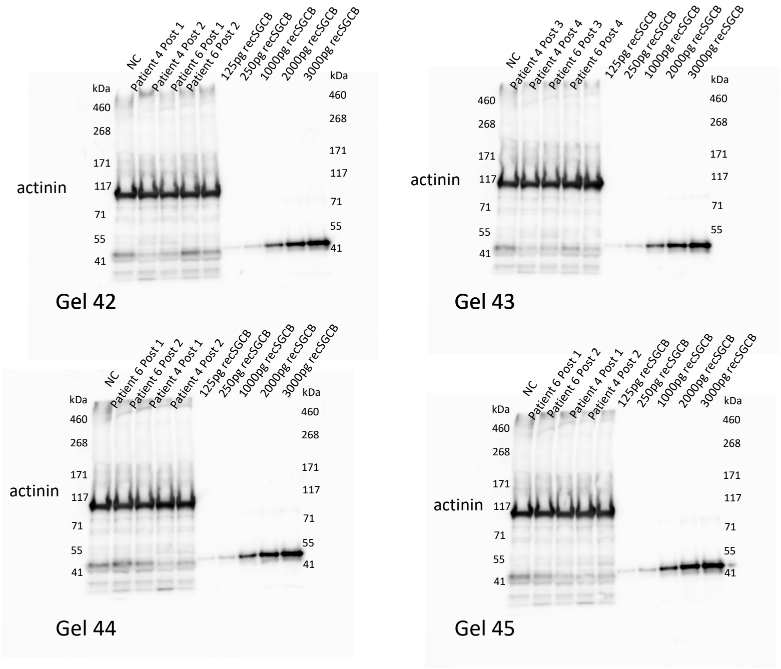

Supplement: Supplementary file 1 — Supplementary Tables 1 and 2 and Figs. 1–3. [file 41591_2023_2730_MOESM1_ESM.pdf]
