# Supplementary material for: Gene therapy with bidridistrogene xeboparvovec for limb-girdle muscular dystrophy type 2E/R4: phase 1/2 trial results
Source: Nat Med. 2024 Jan 4;30(1):199–206. doi: 10.1038/s41591-023-02730-9 (PMC10803256; doi:10.1038/s41591-023-02730-9)
Supplement: Supplementary file 2 — Reporting Summary [file 41591_2023_2730_MOESM2_ESM.pdf]

Corresponding author(s): Louise Rodino-Klapac
Last updated by author(s): Nov 15, 2023

Reporting Summary

Nature Portfolio wishes to improve the reproducibility of the work that we publish. This form provides structure for consistency and transparency in reporting. For further information on Nature Portfolio policies, see our Editorial Policies and the Editorial Policy Checklist.

Statistics

For all statistical analyses, confirm that the following items are present in the figure legend, table legend, main text, or Methods section.

n/a	Confirmed	
☐	☒	The exact sample size (*n*) for each experimental group/condition, given as a discrete number and unit of measurement
☐	☒	A statement on whether measurements were taken from distinct samples or whether the same sample was measured repeatedly
☒	☐	The statistical test(s) used AND whether they are one- or two-sided<br>*Only common tests should be described solely by name; describe more complex techniques in the Methods section.*
☒	☐	A description of all covariates tested
☒	☐	A description of any assumptions or corrections, such as tests of normality and adjustment for multiple comparisons
☐	☒	A full description of the statistical parameters including central tendency (e.g. means) or other basic estimates (e.g. regression coefficient) AND variation (e.g. standard deviation) or associated estimates of uncertainty (e.g. confidence intervals)
☒	☐	For null hypothesis testing, the test statistic (e.g. *F*, *t*, *r*) with confidence intervals, effect sizes, degrees of freedom and *P* value noted<br>*Give P values as exact values whenever suitable.*
☒	☐	For Bayesian analysis, information on the choice of priors and Markov chain Monte Carlo settings
☒	☐	For hierarchical and complex designs, identification of the appropriate level for tests and full reporting of outcomes
☒	☐	Estimates of effect sizes (e.g. Cohen's *d*, Pearson's *r*), indicating how they were calculated

Our web collection on statistics for biologists contains articles on many of the points above.

Software and code

Policy information about availability of computer code

Data collection	The study database is in Medidata Rave EDC System
Data analysis	SAS version 9.4 (SAS Institute Inc., Cary, NC) was used for demographics, safety and efficacy data, and Prism version 5 (GraphPad Software, San Diego, CA) was used for biopsy data

For manuscripts utilizing custom algorithms or software that are central to the research but not yet described in published literature, software must be made available to editors and reviewers. We strongly encourage code deposition in a community repository (e.g. GitHub). See the Nature Portfolio guidelines for submitting code & software for further information.

Data

Policy information about availability of data

All manuscripts must include a data availability statement. This statement should provide the following information, where applicable:

- Accession codes, unique identifiers, or web links for publicly available datasets
- A description of any restrictions on data availability
- For clinical datasets or third party data, please ensure that the statement adheres to our policy

Qualified researchers may request access to the data that support the findings of this study from Sarepta Therapeutics Inc., by contacting medinfo@sarepta.com.

Human research participants

Policy information about studies involving human research participants and Sex and Gender in Research.

Reporting on sex and gender	Findings do not apply to only one sex or gender. No restrictions or requirements were made in study recruitment based on participants identified sex or gender. The identified (self-reported) sex of the participants were 3 male and 3 female. Analysis is descriptive with no sex or gender based analysis performed due to small sample size.
Population characteristics	Age, years, Sex, Height (cm), Weight (kg), BMI (kg/m2), SGCB mutation, Creatine kinase (U/L)
Recruitment	6 LGMD2E subjects with β-SG mutation by DNA testing were enrolled at the study site for this gene transfer study. Subjects could encompass any ethnic, racial, or gender background. Subjects had to have proven muscle weakness to be enrolled. Dr. Mendell recruited all patients. Patient selection criteria include:<br><br>Inclusion Criteria:<br>1. Subjects aged 4 through 15 years, inclusive<br>2. Males or females of any ethnic group<br>3. β-SG DNA gene mutations at both alleles (if genetic testing was completed at a laboratory that is not Clinical Laboratory Improvement Amendments-certified, the testing may be repeated at the discretion of the PI)<br>4. Weakness demonstrated, based on history of difficulty running, jumping, and climbing stairs<br>5. 100MWR test result: ≥ 40% of that predicted for age-, height-, gender-, and weight-matched healthy controls at the screening visit<br><br>Ful inclusion criteria provided in the supplement
Ethics oversight	The study protocol was approved by Nationwide Children's Hospital (NCH) institutional review board. Parents or legal guardians of all patients provided written informed consent before study participation and genetic testing. We followed the Transparent Reporting of Evaluations with Nonrandomized Designs (TREND) reporting guidelines.

Note that full information on the approval of the study protocol must also be provided in the manuscript.

Field-specific reporting

Please select the one below that is the best fit for your research. If you are not sure, read the appropriate sections before making your selection.

☒ Life sciences ☐ Behavioural & social sciences ☐ Ecological, evolutionary & environmental sciences

For a reference copy of the document with all sections, see nature.com/documents/nr-reporting-summary-flat.pdf

Life sciences study design

All studies must disclose on these points even when the disclosure is negative.

Sample size	The sample size of the study was based on enrollment feasibility, and was not based on statistical considerations.
Data exclusions	No data were excluded from analysis
Replication	All analyses generated by SAS (v9.4) were independently QC'd by a second SAS programmer. Independent QCs by SAS programmer and Biostatisticians confirmed the replication of analysis results.
Randomization	There was no randomization in this study. Covariate control was not applicable to this study because the study has a set of inclusion and exclusion criteria to ensure that enrolled patients were appropriate for the evaluation of the study objectives.
Blinding	There was no blinding in this open-label study

Reporting for specific materials, systems and methods

We require information from authors about some types of materials, experimental systems and methods used in many studies. Here, indicate whether each material, system or method listed is relevant to your study. If you are not sure if a list item applies to your research, read the appropriate section before selecting a response.

Materials & experimental systems

n/a	Involved in the study
☐	☒ Antibodies
☒	☐ Eukaryotic cell lines
☒	☐ Palaeontology and archaeology
☒	☐ Animals and other organisms
☐	☒ Clinical data
☒	☐ Dual use research of concern

Methods

n/a	Involved in the study
☒	☐ ChIP-seq
☒	☐ Flow cytometry
☒	☐ MRI-based neuroimaging

Antibodies

Antibodies used

β-sarcoglycan (mouse monoclonal, Leica Biosystems, New Castle, UK; Cat. No. NCL-L-b-SARC)
α-sarcoglycan (mouse monoclonal, Leica Biosystems, New Castle, UK; Cat. No. NCL-L-a-SARC)
γ-sarcoglycan (mouse monoclonal, Leica Biosystems, New Castle, UK; Cat. No. NCL-g-SARC)
δ-sarcoglycan (mouse monoclonal, Leica Biosystems, New Castle, UK; Cat. No. NCL-d-SARC)
Alexa Fluor® Goat anti-mouse IgG (Alexa Fluor™ 594, ThermoFisher Scientific/Invitrogen/Molecular Probes/Cat No. A11032
Antibody

Beta-sarcoglycan
Alpha-sarcoglycan
Delta-sarcoglycan
Gamma-sarcoglycan
Alexa Fluor® Goat anti-mouse igGCohort 1, Day 60

Lot number:
Beta-sarcoglycan Cohort 1, Y2: 6074222, Cohort 2, Day 60: 6070237, Cohort 2, Y2: 6079315
Alpha sarcoglycan Cohort 1, Y2: 6066091 Cohort 2, Day 60: 6066091 Cohort 2, Y2: 6075705
Delta sarcoglycan Cohort 1, Y2: 6074956 Cohort 2, Day 60: 6074956 Cohort 2, Y2: 6088257
Gamma sarcoglycan Cohort 1, Y2: 6074971 Cohort 2, Day 60: 6074971 Cohort 2, Y2: 6088624
Alexa Fluor® Goat anti-mouse igG Cohort 1, Y2: 2069816 Cohort 2, Day 60: 2069816 Cohort 2, Y2: 2192307

Anibodies for Western blot:

Beta-sarcoglycan
Cohort 1, Day 60 NBP1-90300 Novus Biologicals Lot: no information
Cohort 1, Y2 ab83699 abcam lot GR3289209-13
Cohort 2, Day 60 ab83699 abcam lot GR3289209-10
Cohort 2, Y2 ab83699 abcam lot GR3289209-12

Validation

N/A - Commercially available sarcoglycan antibodies, Instructions for Use (IFUs) available at leicabiosystems.com

Clinical data

Policy information about clinical studies
All manuscripts should comply with the ICMJE guidelines for publication of clinical research and a completed CONSORT checklist must be included with all submissions.

Clinical trial registration

NCT03652259

Study protocol

Protocol supplied

Data collection

Center for Gene Therapy, The Research Institute at Nationwide Children's Hospital, Columbus, Ohio, USA. First screening visit with data collection was 10 OCT 2018. Data collection has been ongoing since that date as the study is still active. The most recent data collection was 16 JAN 2023.

Outcomes

Primary Objective:
• To evaluate the safety of SRP-9003
Secondary Objective:
• To quantify expression of β-sarcoglycan (β-SG) in the skeletal muscle of limb-girdle muscular dystrophy, type 2E (LGMD2E) subjects at Day 60 after treatment with SRP-9003

Primary Endpoints
• Incidence of treatment-emergent adverse events
• Incidence of treatment-emergent serious adverse events (SAEs)

Secondary Endpoints
• Change in quantity of β-SG protein expression from Baseline to Day 60, as assessed by Western blot
• Change in quantity of β-SG protein expression from Baseline to Day 60, as assessed by immunofluorescence (IF; IF fiber intensity)
• Change in quantity of β-SG protein expression from Baseline to Day 60, as assessed by immunohistochemistry (IHC) percent β-SG positive fibers (PβSGPF)

Exploratory Endpoints
• Functional outcomes (NSAD score and time function tests)
• Skeletal muscle strength
• Serum CK levels
• Immunogenicity of bidridistrogene xeboparvovec
• Durability of SGCB expression in skeletal muscle
