# Supplementary figures and images for: Gene therapy with bidridistrogene xeboparvovec for limb-girdle muscular dystrophy type 2E/R4: phase 1/2 trial results
Source: Nat Med. 2024 Jan 4;30(1):199–206. doi: 10.1038/s41591-023-02730-9 (PMC10803256; doi:10.1038/s41591-023-02730-9)

Cohort 2, unprocessed western blot – SGCB staining

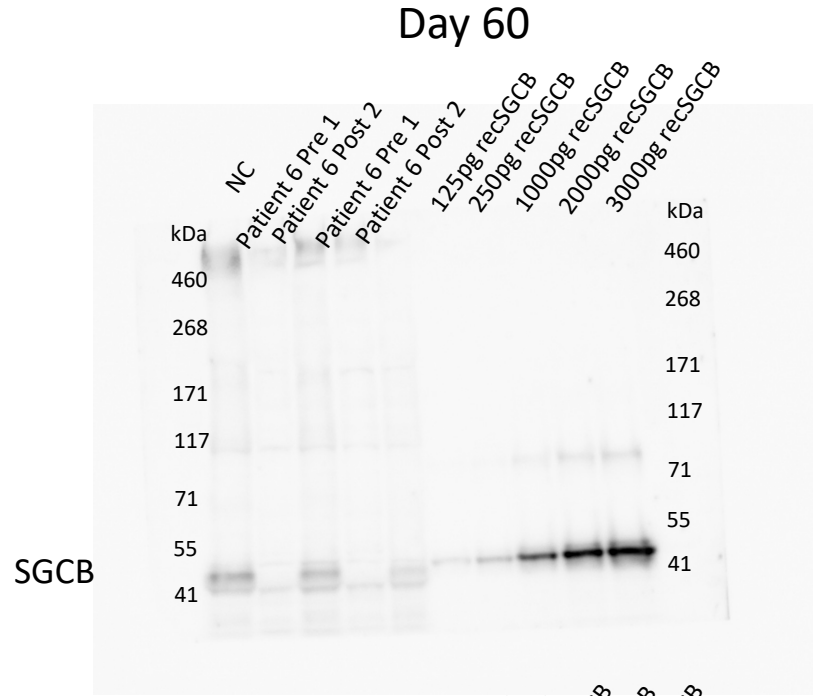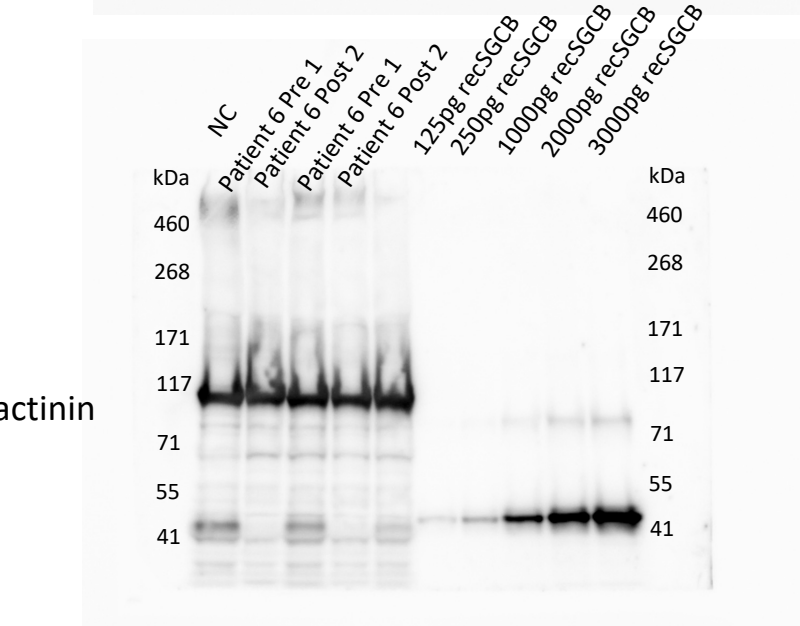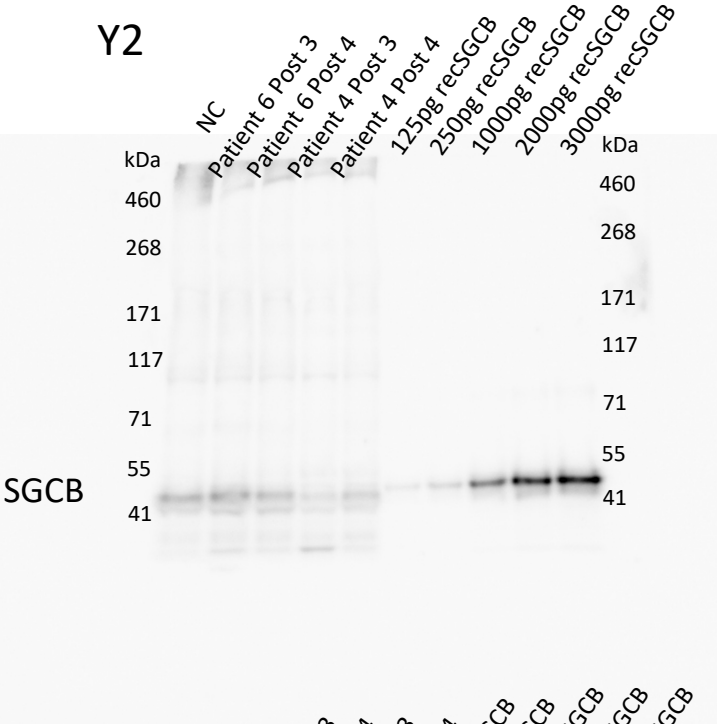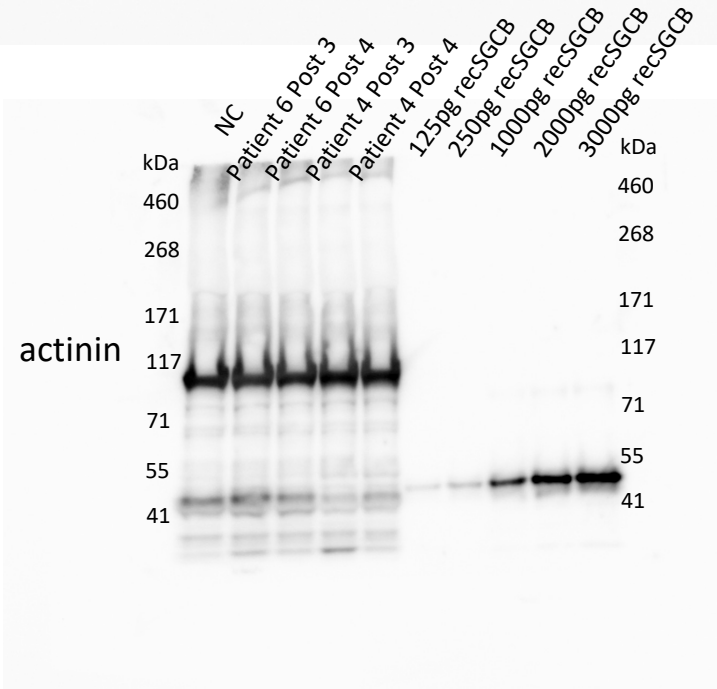

Supplement: Supplementary file 3 — Unprocessed immunoblots used in Extended Data Fig. 2. [file 41591_2023_2730_MOESM3_ESM.pdf]
